# Size-resolved aerosol at a Coastal Great Lakes Site: Impacts of new particle formation and lake spray

**DOI:** 10.1371/journal.pone.0300050

**Published:** 2024-04-04

**Authors:** Megan B. Christiansen, Charles O. Stanier, Dagen D. Hughes, Elizabeth A. Stone, R. Bradley Pierce, Jacob J. Oleson, Sherrie Elzey

**Affiliations:** 1 Department of Chemical and Biochemical Engineering, University of Iowa, Iowa City, Iowa, United States of America; 2 Department of Chemistry, University of Iowa, Iowa City, Iowa, United States of America; 3 Space Science and Engineering Center, University of Wisconsin-Madison, Madison, Wisconsin, United States of America; 4 Department of Biostatistics, University of Iowa, Iowa City, Iowa, United States of America; 5 TSI Incorporated, Shoreview, Minnesota, United States of America; University of Birmingham, UNITED KINGDOM

## Abstract

The quantification of aerosol size distributions is crucial for understanding the climate and health impacts of aerosols, validating models, and identifying aerosol sources. This work provides one of the first continuous measurements of aerosol size distribution from 1.02 to 8671 nm near the shore of Lake Michigan. The data were collected during the Lake Michigan Ozone Study (LMOS 2017), a comprehensive air quality measurement campaign in May and June 2017. The time-resolved (2-min) size distribution are reported herein alongside meteorology, remotely sensed data, gravimetric filters, and gas-phase variables. Mean concentrations of key aerosol parameters include PM_2.5_ (6.4 μg m^-3^), number from 1 to 3 nm (1.80x10^4^ cm^-3^) and number greater than 3 nm (8x10^3^ cm^-3^). During the field campaign, approximately half of days showed daytime ultrafine burst events, characterized by particle growth from sub 10 nm to 25–100 nm. A specific investigation of ultrafine lake spray aerosol was conducted due to enhanced ultrafine particles in onshore flows coupled with sustained wave breaking conditions during the campaign. Upon closer examination, the relationships between the size distribution, wind direction, wind speed, and wave height did not qualitatively support ultrafine particle production from lake spray aerosol; statistical analysis of particle number and wind speed also failed to show a relationship. The alternative hypothesis of enhanced ultrafine particles in onshore flow originating mainly from new particle formation activity is supported by multiple lines of evidence.

## Introduction

Aerosols play an important role in the effects of air pollution on human health, cloud interactions, and climate change. Aerosols have direct effects on the climate (e.g. scattering and absorbing solar radiation) and indirect effects through cloud microphysics and albedo [1, 2]. Aerosols classified as fine particulate matter (PM_2.5_; aerosols ≤ 2.5 μm aerodynamic diameter) are a concern to the human population sensitive to respiratory illnesses because of their ability to deposit in the airways and lungs [3, 4]. Thus, PM_2.5_ is a criteria air pollutant monitored and regulated by the Environmental Protection Agency (EPA) through the National Ambient Air Quality Standards (NAAQS). However, many climate and health effects of aerosol particles are strongly influenced by particle size; thus, measurement of aerosol size distribution in concert with metrics such as PM_2.5_ gives a much more complete picture of aerosol processes. For example, ultrafine aerosol particles (with diameters less than 100 nm) account for a significant fraction of inhaled aerosols, particularly if the respirable dose is weighted by particle number or surface area [5, 6]. These aerosols have very little volume or mass and are therefore missed by measuring solely PM_2.5_ concentrations.

Two potentially important sources of ultrafine aerosol particles (UFP) in the Great Lakes region of the U.S. that can be constrained by coastal size-resolved measurements include atmospheric new particle formation (NFP) and growth and UFP from breaking freshwater waves.

New particle formation (NPF) is a controlling factor for aerosol number concentrations, cloud condensation nuclei concentrations, and aerosol cloud interactions [7, 8]. The process of NPF and growth is detected ubiquitously but with spatiotemporal variation in details such as frequency and strength, as well as dependence on atmospheric conditions [9–13]. Long-term shifts in NPF precursors are likely influencing counts and distributions of UFP in many locations [14]. For example, a 70% reduction from 2001 to 2017 in urban UFP was reported in one US study and attributed to reductions in SO_2_ concentrations [15] and similar trends have been documented in other US urban regions [14].

Studies of NPF and growth have been refined as the lower size limit of sizing instrumentation has decreased, mainly through advancements in condensation particle counter design. Widespread detection down to 3 nm was enabled by the TSI 3025 “ultrafine” condensation particle counter (CPC), which was based on the design of Stolzenburg and McMurry [16]. Further refinements have enabled increasingly detailed study of charged and neutral aerosols and clusters as smaller sizes. For charged aerosols, instruments such as the electrical aerosol spectrometer are used; particles pass through two unipolar charging units (diffusion and field charging) [17]. In the Air Ion Spectrometer (AIS), naturally charged particles are split between two oppositely charged mobility analyzers [18]. The lower limit of detectable size for neutral aerosols has been extended to below 2 nm using pre-growth chambers [19, 20] and the Neutral cluster AIS [21]. Specifically, a diethylene glycol (DEG) UCPC is placed inline of a scanning mobility particle sizer (SMPS) as a “pre-growth” step, and then particles are passed to a butanol CPC to further grow the particles to sizes detectable by light scattering [22]. This design was commercialized as a sub 3-nm SMPS by TSI and used during LMOS 2017. The instrument is described further in Aerosol Size Instrumentation section.

Sea spray aerosols (SSA) are known to be large contributors to the atmospheric aerosol population [23] and are widely included in Photochemical Grid Models (PGM) [24–26]. The corollary in freshwater is Lake Spray Aerosol (LSA). Aerosol generation studies using synthetic wave or spray generation report differences in size distributions and chemical composition between SSA and LSA driven by differences in ion concentrations and composition of the source water [23, 27]. LSA may be an important contributor to aerosol populations, aerosol-cloud interactions, and they may be important vectors of toxins during inland harmful algal blooms [28, 29]. Modeling suggests that LSA could influence particle number by 20% [30] and PM mass by 5–25% [31]. Furthermore, LSA may modulate the rates of other aerosol microphysical processes such as deposition, gas-particle partitioning, and NPF [31]. However, their strength, frequency, and representation in PGM are highly uncertain.

There is only one observational constraint on UFP from LSA. During the CABINEX campaign, flight-based aerosol size distributions over Lake Michigan showed that UFP, with a mode of 30 nm, were prominent over the lake especially during time periods of high wind speeds associated with breaking waves [32]. Similarly, during LMOS 2017 a significant peak with mode of 38 nm in the aerosol size distributions was present following lake breeze events when compared to pre lake breeze arrival [33]. This “more UFP in the lake breeze” signal that we previously reported, coupled with broad interest in LSA for public health and climate, motivated a closer look at whether LMOS 2017 can observationally confirm an ultrafine mode in LSA.

Here we report the size-resolved aerosol measurement taken during the Lake Michigan Ozone Study 2017 (LMOS 2017), and use them to inform questions about relative importance of ultrafine particle sources in the region. LMOS 2017 was a multi-site collaborative field campaign developed to gather high spatio-temporal resolution data in support of ongoing efforts for improvement of regional air quality [34]. The campaign provided extensive observational datasets regarding ozone, its precursors, particulate matter, and meteorology associated with ozone events through a combination of airborne, ship, mobile lab, and fixed ground-based sites. The overarching goal of LMOS was to investigate ozone formation and transport; aerosol measurements during LMOS were conducted to support source apportionment and site characterization. Photochemical Grid Models (PGM) were used for forecasting and post-campaign analysis, and ground-based, aircraft, and satellite remote sensing products have been integrated into LMOS analyses.

During LMOS 2017, aerosol measurements were conducted primarily at the Zion, IL ground site, 67 km north of Chicago. This paper builds on previous publications that have discussed aerosol and particle-phase measurements from Zion. Mean daytime concentrations of particle number (5711 cm^-3^), PM_2.5_ (6.4 μg m^-3^), and PM_10_ (8.3 μg m^-3^) were reported in Doak et al. [35] together with a comprehensive site characterization for Zion. Doak et al. [35] also used particle size distribution measurements in positive matrix factorization source apportionment, and to quantify enhancement in ultrafine particles within a few minutes of passage of diesel locomotives, on the rail line 0.54 km from the site. Hughes et al. [36] reported PM_2.5_ speciation, dominated by organic matter (average of 59%), and showed significant variation in chemical composition and regional origin of PM_2.5_ during each of the three high ozone event periods. Wagner et al. [33] presented detailed characterization of the lake breeze behavior during LMOS 2017, quantifying the well-known sharp changes in wind direction, temperature, water vapor, and stability at time of lake breeze arrival. Furthermore, Wagner et al. [33] showed that lake breeze arrival was also associated with a sudden and statistically significant increase in UFP, followed by gradual increases in PM_2.5_ and aerosol backscatter [33].

Previous studies deployed impactors to study aerosol size distribution and composition around the Great Lakes. During the LMOS 1991 field campaign, the Lake Michigan Urban Air Toxics Study (LMUATS) measured several aerosol air toxin species at a ground station in downtown Chicago and aboard a research vessel stationed offshore of Chicago on Lake Michigan [37]. The Atmospheric Exchange over Lakes and Oceans (AEOLOS) occurred during July 1994 and January 1995 in urban Chicago, IL and over southern Lake Michigan [38]. In these studies, the focus was better understanding of the atmospheric toxic chemicals and trace metals that affect the Lake Michigan ecosystem. Thus, mass distributions of particulate air toxics were determined based on the size cuts of the specific impactors used. Previous measurements of size distributions have been reported near Lake Michigan but not directly at the shoreline. Measurements at 25 km away from the lakeshore, at the University of Michigan Biological Station in northern Michigan, have been reported in Kanawade et al. [12] and Gunsch et al. [39]. Kanawade et al. [12] measured for 34 days in summer 2009 in the 3–800 nm range. Gunsch et al. [39] measured for 45 days in summer 2014 in the 11–603 nm range.

While previous LMOS 2017 publications have used portions of the aerosol size distribution and particle count data measured at Zion, in this paper we present the comprehensive result of the full aerosol size distribution and its temporal variation, merged across three sizing instruments. The details of field deployment, data processing, and quality assurance of the aerosol sizing instrumentation are reported here. We compare a standard SMPS and the novel DEG-boosted CPC / SMPS system in their overlapping size range (12–32 nm). We furthermore report comparison to the independently measured particle number from a CPC, to filter-based aerosol mass, and to aerosol optical depth measured by AERONET at Zion. These are used in conjunction with wind and wave measurements to attempt to observationally confirm the impact of LSA on ultrafines during LMOS 2017. This comprehensive report and analysis of the aerosol number size distribution measured at Zion, IL during LMOS 2017 is meant to inform aerosol modeling and measurement studies motived by health and climate effects, evaluate the novel DEG-boosted CPC / SMPS, document the relationship between PM_2.5_ and AOD and the location, provide insight into the processes controlling the aerosol distribution, and provide a rare field assessment of the impact of LSA on ultrafine aerosols.

## Methods

### Campaign and site description

LMOS 2017 occurred from May 22 to June 22 2017. The campaign employed two aircraft, ship, mobile labs, two enhanced-monitoring sites (Spaceport Sheboygan, WI and Zion, IL), and various supplemental remote sensing systems. Further details and an overview of LMOS 2017 have been described in Stanier et al. [34]. The Zion site (42.468 N, 87.810 W) was collocated with an Illinois AQS monitoring station (AQS ID 17-097-1007) inside the Illinois State Beach Park. The site is 900 m inland due west from the lake shore with an active rail line and main arterial roadway 540 m and 1.3 km due west, respectively [35]. Field access was approved by the Lake Michigan Biological Station, The Illinois Beach State Park, and the Illinois Environmental Protection Agency, which all had overlapping oversight of the field station and its supporting infrastructure.

### Instrumentation

#### Aerosol size instrumentation

The University of Iowa deployed several instruments during the LMOS campaign at the Zion, IL ground site to measure the full particle size distribution (PSD). A summary of the variables measured, time resolutions, and sampling instruments are listed in Table 1 with aerosol instrument flow diagrams in S1 Fig in S1 File. Three separate inlets were used for particle sizing and counting equipment.

**Table 1:** Zion site instrument information reported in this work.

Instrument	Measurement	Sampling Frequency
**TSI 1 nm SMPS**	PSD 1–32 nm	2 min
**TSI Std. SMPS**	PSD 12–562 nm	2 min
**TSI APS 3321**	PSD 542 nm– 10 μm	2 min
**TSI CPC 3025**	Total particle number, lower limit 3 nm	2 min
PM_2.5_ medium-volume filter samplers	PM_2.5_ mass, elemental carbon, organic carbon, inorganic ions (sodium, potassium, magnesium, calcium, ammonium, chloride, nitrite, nitrate, sulfate), select metals, molecular organic tracers	12 hr
**AERONET**	Aerosol optical depth	Varying

The first inlet, designed for high particle transmission of 1–10 μm particles, supplied an Aerodynamic Particle Sizer (APS, TSI 3321). The inlet included no bends and was dried with a diffusion dryer (TSI 3062) and equipped with a size-selective cyclonic inlet (PM_10_, BGI). The APS reported data in channels ranging from 0.542 to 20 microns; in this work, we report results from 0.542 to 10 microns (aerodynamic diameter); as described below, these were later converted to electrical mobility diameters to create a full size distribution across all of the instruments.

A second inlet was shared by a scanning mobility particle sizer (SMPS, TSI 3936L81) and an independent butanol condensation particle counter (CPC, TSI 3025) with a nominal 3 nm lower size cutoff for particle detection. The standard SMPS was equipped with a long DMA (TSI DMA 3081), Kr-85 neutralizer, diffusion dryer (TSI 3062), and water CPC (TSI 3785) with inlet and sheath flows of 1 and 4 LPM, respectively. The sheath air flow was further dried with inline silica gel absorbent. We report particle counts at sizes ranging from 12.2 to 552.3 nm in this work, and refer to this as the “SMPS” result.

A third inlet was used by the TSI 1 nm SMPS (TSI 3938E77), equipped with a 1 nm DMA column (TSI 3086), soft x-ray neutralizer (TSI 3088), diethylene glycol nano enhancer (TSI DEG enhancer 3777), and butanol CPC (TSI 3772) with inlet and sheath flows of 2.5 and 25 LPM, respectively. This inlet was kept short in length (15 cm) and was not dried; these choices were implemented to maximize particle transmission efficiency. We report results from 1.02 to 32.0 nm in this work, and refer to this as the “1 nm SMPS” result.

Relative humidity probes (RH, Sensirion sensors, SHT75) continuously monitored the sampling lines at multiple points of the SMPS and APS instruments.

#### Additional instrumentation and data availability

PM_2.5_ was collected by medium-volume integrated aerosol filters (3000B, URG Corporation) onto 47 mm Teflon filters at a flow rate of 90 liters per minute twice daily and the composition was analyzed post campaign as described in Hughes et al. [36]. The Wisconsin Department of Natural Resources also measures PM_2.5_ at Chiwaukee Prairie (AQS ID 55-059-0019) by beta attenuation monitors, located 4 km north of the Zion site. AERONET level 2 data for aerosol optical depth (AOD) and spectral deconvolution algorithm aerosol fractions were downloaded from www.aeronet.gsfc.nasa.gov/index.html. The AERONET was installed at Zion site from June 4 to June 21 2017. AOD was interpolated to 550 nm using an angstrom exponent

$$\tau_{\lambda_{550}}={\tau_{\lambda_{500}}(\frac{\lambda_{550}}{\lambda_{500}})}^{-\alpha }$$ 1)

where λ_500_ is the wavelength 500 nm, λ_550_ is the wavelength 550 nm, τ is AOD at the specified wavelength, and α is the angstrom exponent (440–870 nm) as reported by AERONET.

A complete list of campaign instrumentation is available in the supplemental information to Stanier et al. [34], with additional details for the Zion site in Doak et al. [35]. Data are available for public download at the NASA repository [40].

A dataset of estimated monthly mean PM_2.5_ (V5.GL.02), with a spatial resolution of 0.01°x 0.01°, publicly available from the Atmospheric Composition Analysis Group at Washington University in St. Louis was used for discussion purposes in the Spatial Context and Air Quality Implications (https://sites.wustl.edu/acag/datasets/surface-pm2-5/#versioninfo). The methods of estimation are detailed in van Donkelaar et al. [41].

The Wilmette Buoy, IL (Station 45174; 42.135 N, 87.655 W) is located 7.5 km offshore of Glencoe, IL and 39 km southeast of Zion site. The dataset was downloaded from the National Data Buoy Center (https://www.ndbc.noaa.gov/historical_data.shtml).

### Data quality assurance and analysis

Routine flow rates and leak checks on each aerosol instrument were performed every three days and following each exchange of silica absorbent. When the RH values exceeded 50% (aerosol inlets) or 20% (SMPS recirculating sheath flow), fresh absorbent was exchanged in. Daily site logs were kept for each instrument and overall site observations including current weather, activity around site, interior trailer condition, and personnel arrival and departure times.

Post campaign quality assurance consisted of flagging data using NARSTO categories (S1 Table in S1 File) [42]. This included periods of known invalid data such as in-field instrument downtime, visual inspection for physically unrealistic data, and visual inspection for exceptional events that coincided with daily site logs. The SMPS was tested with certified polystyrene latex spheres (100 nm PSL spheres, Thermo Scientific) to ensure the accuracy of the particle size measurements of the SMPS. The particle size distributions (APS, SMPS, and 1 nm SMPS) were adjusted for diffusional, inertial, and gravitational losses by calculating transmission efficiency curves (S2 Fig in S1 File) for each instruments’ respective inlet [42]. The 1 nm SMPS curve (blue line, S2 Fig in S1 File) includes corrections for the neutralizer and instrument diffusional losses. The APS particle diameters were shifted from aerodynamic diameter to electrical mobility diameter by Eq 2 [43]

$$D_{p}=D_{a}\sqrt{\chi \frac{\rho_{o}}{\rho_{p}}}$$ 2)

where D_p_ is the electrical mobility diameter, D_a_ is the aerodynamic diameter, ρ_o_ is the reference density (1.0 g cm^-3^), χ is the shape factor, and ρ_p_ is the calculated particle density.

The three instruments’ distributions were merged in MATLAB to create the overall size distribution for the entire campaign. In the overlap range between 12 and 32 nm, the SMPS was given preference, because a) the SMPS was subjected to post campaign QA/QC checks at the University of Iowa, including monodisperse PSL spheres while the 1 nm SMPS was not and b) this facilitated comparison to previous Midwestern field deployments of the SMPS [44–46]. When the SMPS was not available, the 1 nm SMPS was used in this size range. In the SMPS-APS overlap region (542–562 nm), the two instruments were averaged. The merged distribution is referred to as the particle size distribution (PSD).

The CPC number concentration was corrected by Eq 3

$${CPC}^{*}=CPC*\frac{{PSD}_{tc}}{{PSD}_{tc,CPC}}$$ 3)

where PSD_tc_ is the PSD total number concentration, PSD_tc,CPC_ is a lower total number based on the measured (and loss corrected) PSD adjusted downward per the CPC transmission efficiency (S2 Fig in S1 File, yellow line); CPC is the measured CPC number concentrations, and CPC* is the corrected CPC number concentrations. The CPC was only corrected during the 2-min time periods in which the full size distribution was available.

Due to variation in instrument uptime across the three sizing instruments, there existed periods where the full merged size distribution (1.02 to 8671 nm) had missing sections. For example, if the APS was down, the size distribution was only available from 1.02 to 562 nm. In such cases, imputation was done to fill missing portions of the size distribution. Imputation was only used to fill in portions of the distribution that were low (i.e., the APS contribution to number, or the 1-nm SMPS contribution to volume). The two-step procedure for this was as follows. In step 1 of the procedure, for each 2-minute time period with any data gap, a distribution of possible gap-filled values was created using the ratio

$$P_{i}=P_{n,j}+P_{x,i}$$ 4)

$$P_{x,i}=\frac{P_{n,i}*P_{x,j}}{P_{n,j}}$$ 5)

where P is the statistic (number, surface area, volume) in question, i is the time period of the gap, j is the time index of a distribution with no missing data, x refers to bins without data for hour i, and n refers to bins where data is present for hour i. For each missing statistic, there were 12,182 values in the distribution, corresponding to the 2-min periods with no missing data (406 hours). In step 2, the median of the distribution of P_i_ values was used as the gap-filled value if two tests were met. The first test was that the new (gap-filled) value did not increase relative to the non-gap-filled value by more than 5%. The second was that the coefficient of variation of the distribution of the possible gap-filled values (P_i_) was small (less than 0.03). The first test made sure that the influence of imputation on the overall statistic (number, surface area, volume) was minor. The second test rejected cases for which the size distribution in the missing bins varied considerably in time, making the gap filling uncertain. Four sensitivity tests were performed to see the impact of this imputation or gap-filling technique. The first two tests involved filling in missing 1-nm SMPS contributions to mass with zero, and with the mean of the available 1-nm SMPS data, respectively. The second two tests involved filling in missing APS contributions to number with zero, and with the mean of the available APS data. PM_2.5_ mass and total number changed by less than 0.05% across the sensitivity tests.

Particle density is required for shifting between mobility and aerodynamic diameters (Eq 2). Density is also used to convert from the measured volume distribution to particle mass for intercomparison to filter-based mass from Hughes et al. [36] and to AQS network measurements, which were done at nearby sites via beta attenuation monitoring. A density of 1.33 g cm^-3^ was used based on collocated particle composition measurements [36] with densities of the major aerosol components as in Lee et al. [47]. Ammonium, nitrate, sulfate, and organic matter made up 87% of the PM_2.5_ mass on average. The ammonium nitrate (AN, ρ = 1.72 g cm^-3^), ammonium sulfate (AS, ρ = 1.79 g cm^-3^), and organic material (OM, ρ = 1.2 g cm^-3^) were present at relative mass fractions of 0.06, 0.25, and 0.69, respectively. A shape factor of 1 was assumed.

We subjected all 2-min data of wind speed and particle number with onshore flows and wind speeds in excess of 2 m/s, to regression analysis to evaluate existence and quantify strength of an LSA UFP contribution. Due to strong serial autocorrelation, the regression accounting for autocorrelation with an autoregressive function of order 1 on the residuals. A log transformation was used to better approximate normality because particle number values were right skewed.

## Results

### Particle number time series

**Fig 1:**
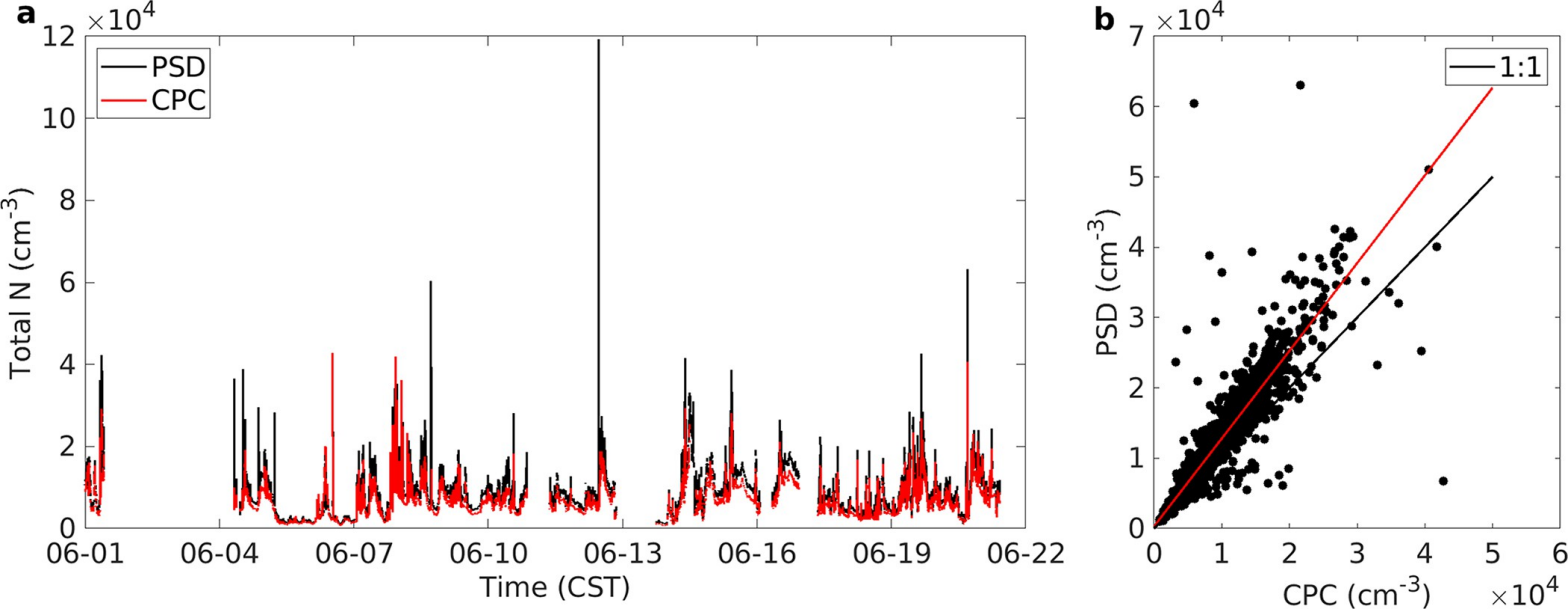
Timeseries (a) of CPC (red) and PSD (black) and resulting scatter plot (b) of 2 min data from June 1 –June 22, 2017. Instrument size cutoffs and loss corrections as reported in text. In (b) the black line is 1:1 and red line is linear regression where the slope is 1.25.

The time series of the particle number concentration from June 1–22, 2017 is shown in Fig 1. Two measurements are shown and intercompared. One is the total number from the PSD (3–8671 nm) and the other is for the stand-alone CPC, which has a nominal lower size limit of 3 nm. Both measurements were corrected for estimated particle losses, averaged to a common 2-minute interval, and synchronized to a common time basis. The period shown lacks the period May 22 –May 31 because the stand-alone CPC did not begin operation until June 1.

The mean concentrations from the two instruments were 8485 and 6469 cm^-3^, respectively. These concentrations are typical for rural and peripheral urban locations. The time series allows visualization of the temporal dynamics (intense peaks, often of short duration and several times per day, creating a skewed distribution), peak concentrations, and instrument downtime. The high correlation between the two entirely independent instruments (Fig 1B, Pearson r = 0.93, index of agreement of 0.91) serves as a quality check on the instruments and on the post-campaign data processing. The difference between the methods, with the PSD-based number concentration as approximately 25% higher than the CPC is well within expected margins of error, considering variation from instrument to instrument, the independent inlets used, and uncertainty in particle loss corrections.

### Particle size distribution

**Fig 2:**
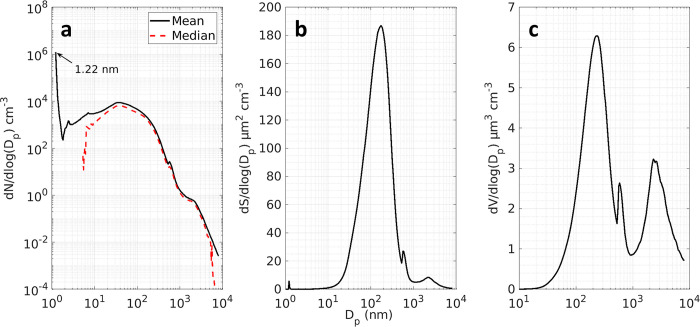
Arithmetic mean of number (a), surface area (b), and volume (c) distributions for the entire campaign period. Solid lines are means and the dashed line is the number distribution median. Calculations of surface area and volume based on a spherical particle assumption.

The grand average size distributions measured during LMOS 2017 are shown in Fig 2. The number distribution was unimodal with a mode at 40 nm and a large peak below 3 nm due to influence on the mean of infrequent, but high particle counts in the 1–10 nm range. The surface area and volume distributions were bimodal. The modes of the surface area distribution were at 173 nm and 2.22 μm, respectively. The modes of the volume distribution were at 223 nm and 2.66 μm, respectively. The first modes of both moments are within the accumulation mode and the second modes are in the coarse mode. Similar distributions have been measured at other rural sites with urban impacts such as Bondville, IL [11]. Discontinuities at 32 nm in the number distribution are due mainly to the different instrument uptime of the 1 nm SMPS and the SMPS. The discontinuity at (560 nm) in the surface and volume distribution reflect different instrument uptime of the SMPS and APS, as well as differences in measured size distribution intensity at the overlap sizes. The volume distribution is consistent with the high fine mode fraction (0.72) reported in the collocated AERONET AOD measurement.

A 40 nm mode in the number distribution suggests a nearby source of nuclei and Aitken mode particles. Visual inspect of the time evolution of the number distribution (discussed below) indicate that contributions from both primary sources and NPF and growth events are driving the number distribution at sizes below 50 nm [12].

### Reconstructed PM mass from the particle size distribution

**Fig 3:**
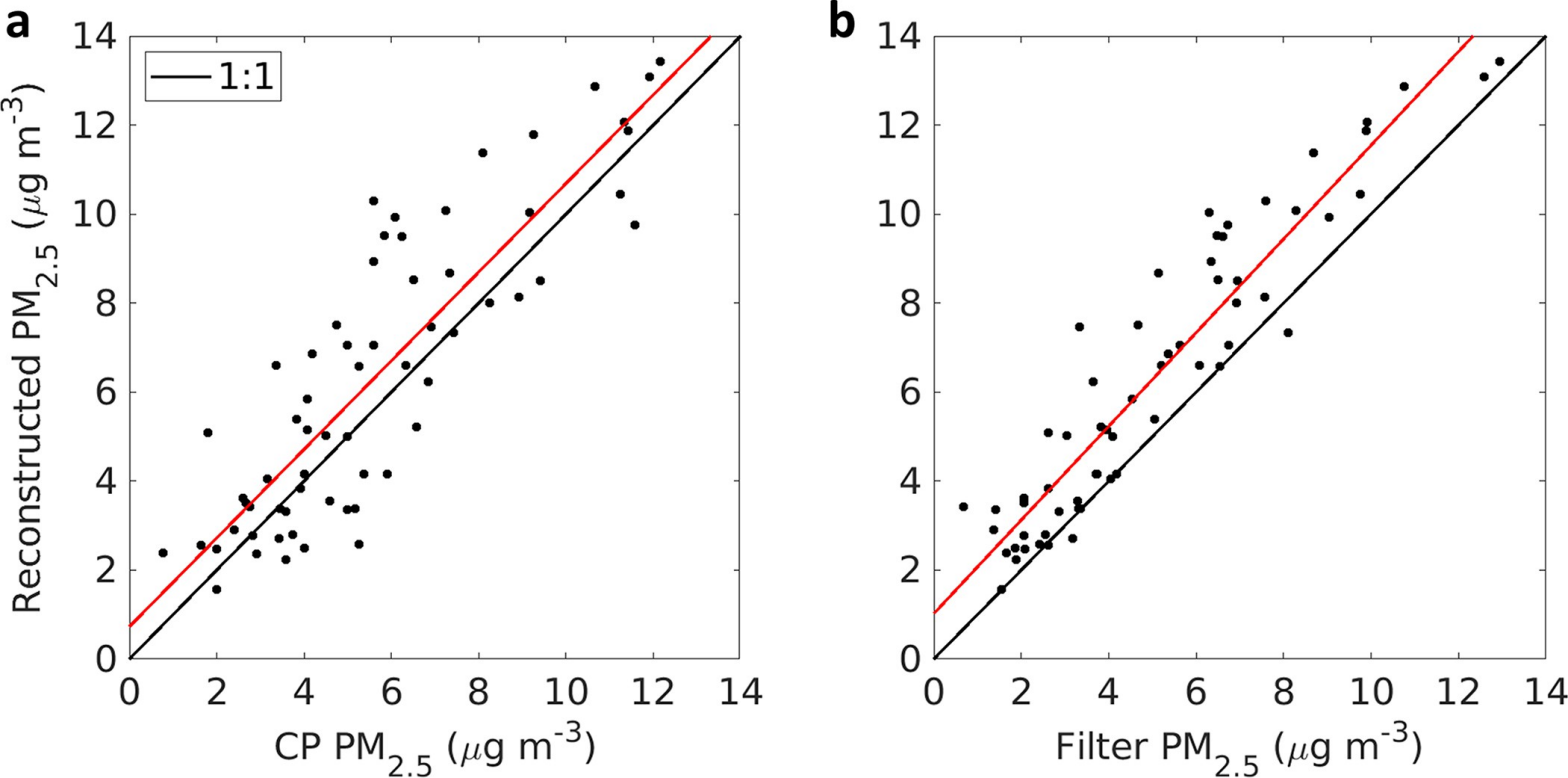
Reconstructed PM_2.5_ at Zion compared to (a) Chiwaukee Prairie BAMS, and (b) Zion gravimetric filters. Black lines are 1:1 and red lines are linear regression where the slopes are (a) 0.996 and (b) 1.053.

The PM mass inferred from the PSD and the aerosol density and shape assumptions (which we hereafter refer to as the reconstructed PM mass) averaged 7.9 μg m^-3^ (PM_10_) and 6.4 μg m^-3^ (PM_2.5_). This indicates that the site has low levels of PM_2.5_ compared to the National Ambient Air Quality Standard of 12 μg m^-3^. These compare well to integrated PM_2.5_ measurements collocated at Zion (5.2 μg m^-3^), and at the nearby Chiwaukee Prairie AQS monitor (6.0 μg m^-3^). Comparison scatterplots (Fig 3), fall close to the 1:1 line. For comparison to 12-h average concentrations, any period missing more than 50% of the PSD was excluded. Correlation coefficients (r) relative to Chiwaukee Prairie and Zion filters were 0.86 and 0.94, respectively. On average the reconstructed mass was higher than that on the filters, but well within expected ranges given potential artifacts in mass-based techniques coupled with uncertainties in particle shape, water content, and density. Decreased correlation at the Chiwaukee Prairie relative to Zion is expected due to the 4 km separation distance, and the likelihood of different local sources and slightly different impacts of from regional transport. This intercomparison adds to the quality assurance of the instrumentation and the post-campaign processing, and allows confident use the 2-min data on surface area, particle volume, and particle mass, to establish temporal details unobservable through the less time-resolved instruments.

### Mass and aerosol optical depth temporal variation

**Fig 4:**
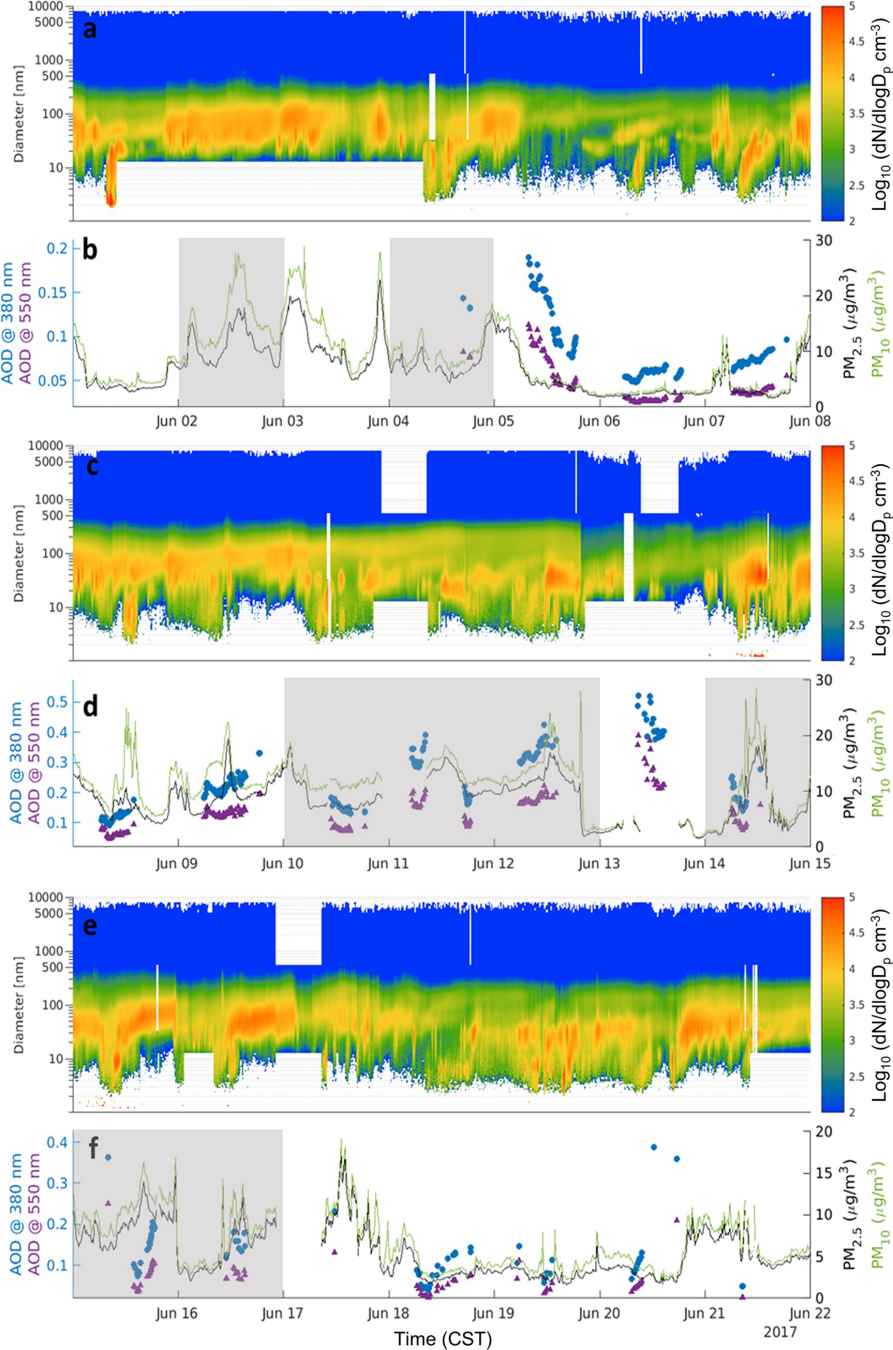
Selected aerosol variables for June 1–7 (a and b), June 8–14 (c and d), and June 15–21 (e and f) averaged to 10 min. The time series of the aerosol number size distribution is shown in panels a, c, and e. The time series of reconstructed PM_2.5_ and PM_10_ mass, and AOD at 380 and 550 nm, are shown in panels b, d, and f. Gray shaded regions represent high ozone event periods.

Temporal evolution of the number PSD together with reconstructed PM_2.5_ and PM_10_ mass are shown in Fig 4 for the period June 1 to June 21. Also shown are the Level 2 AOD values reported by the AERONET station deployed at Zion during the field campaign. Expanded figures showing additional pollutants are in S1 File.

These figures show typical temporal dynamics of the site, and may be of use in adding context in related analyses (i.e., ozone episodes, ozone precursor variation, lake spray aerosol, primary source impacts, remote sensing, meteorology, etc.). Rapid drops in PM_2.5_ and PM_10_ can be seen on June 16 and June 21, where PM drops from above 10 μg m^-3^ to below 5 μg m^-3^ in just a few minutes, due to a change in air mass. The AOD at 380 nm (mean of 0.155) is considerably higher than the AOD at 550 nm (mean of 0.084), consistent with a fine mode dominated aerosol size distribution. Temporal gaps in the AOD are due to AOD only being reported during cloud-free daylight hours.

Periods of elevated PM_2.5_ (above about 15 μg m^-3^) are of interest because they are strong enough to be detected with aerosol remote sensing and may indicate local sources, long-range transport of wildfire smoke (and associated ozone precursors), and/or long-range transport of aged airmasses with high secondary organic aerosol loadings [36]. These can interact differently with local circulations such as the lake breeze, and have different implications for interpreting ozone episodes. Thus, distinguishing between these different particle sources is important, and is typically accomplished through a combination of particle chemistry measurements, satellite imagery, and remotely sensed cloud and aerosol properties.

**Fig 5:**
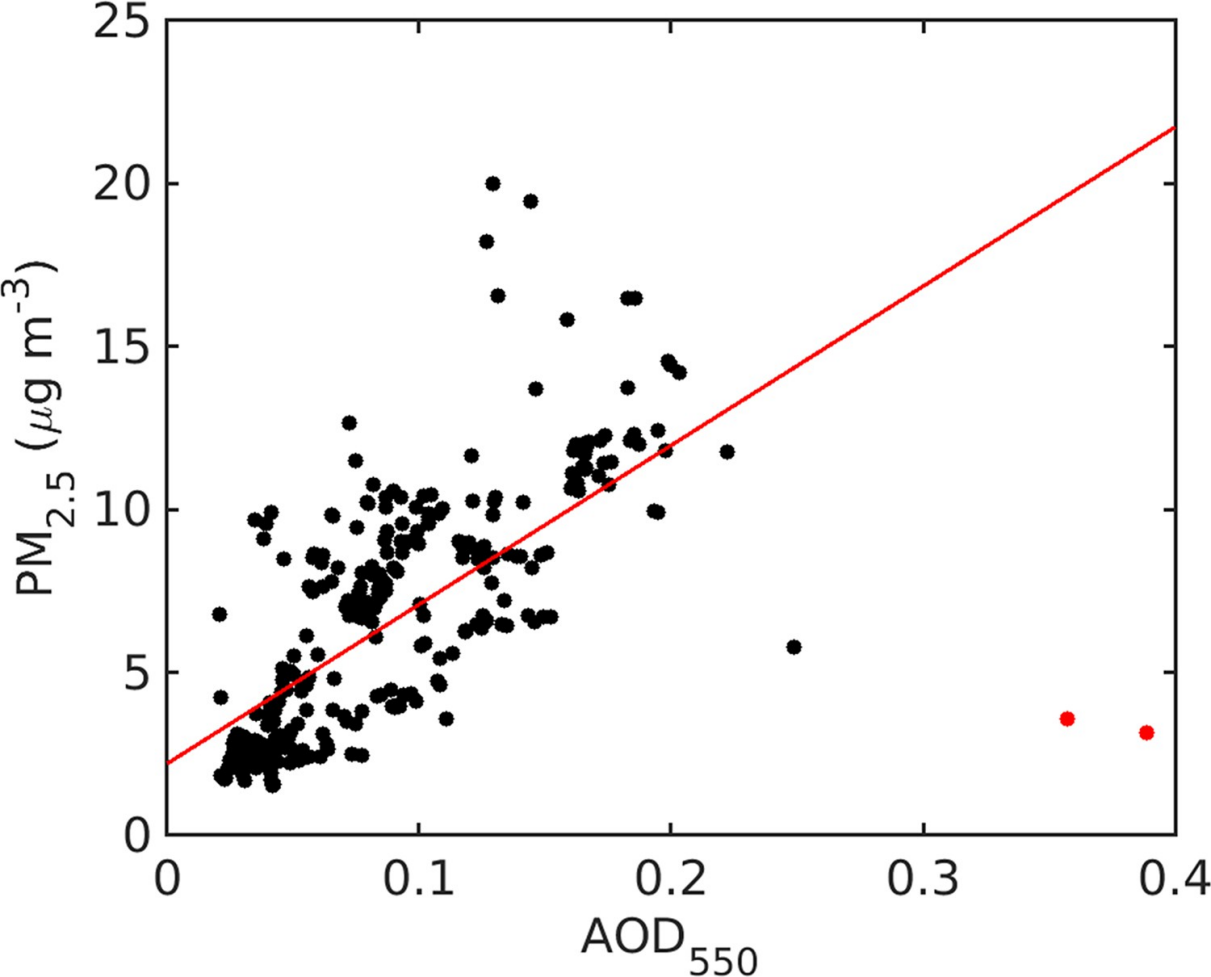
AOD_550_ compared to 2 min averages of the reconstructed PM_2.5_. The linear regression line (red) was calculated excluding the June 13 data (red dots) due to likely cirrus contamination.

The LMOS 2017 dataset adds to the ability to interpret remote sensing in the region by establishing a PM_2.5_/AOD_550_ ratio of 82.4 with a correlation (r) of 0.69, as shown in Fig 5. Periods with PM_2.5_ above 15 μg m^-3^ and with coincident AERONET AOD measurements occurred on June 9 and June 12. The period with highest AOD (June 13) did not have APS measurements necessary for a reconstructed mass, and thus a comparison of ground based PM_2.5_ and the AOD could not be performed. Several hours of June 13 are also not available from the PM_2.5_ recorded at Chiwaukee Prairie. The cloud screening techniques used for level 2 AOD data can be contaminated by fair weather cirrus clouds. GOES-R retrievals showed cirrus cloud over Zion during the time periods of AOD > 0.3 on June 13. Therefore, we highly suspect cloud contamination in the June 13 AERONET AOD; those data were excluded in the regression of Fig 5 (red data points). Distributions of PM_2.5_, PM_10_, coarse PM, and AOD can be found in S1 File.

### Inter-comparison of 1 nm SMPS and standard SMPS

**Fig 6:**
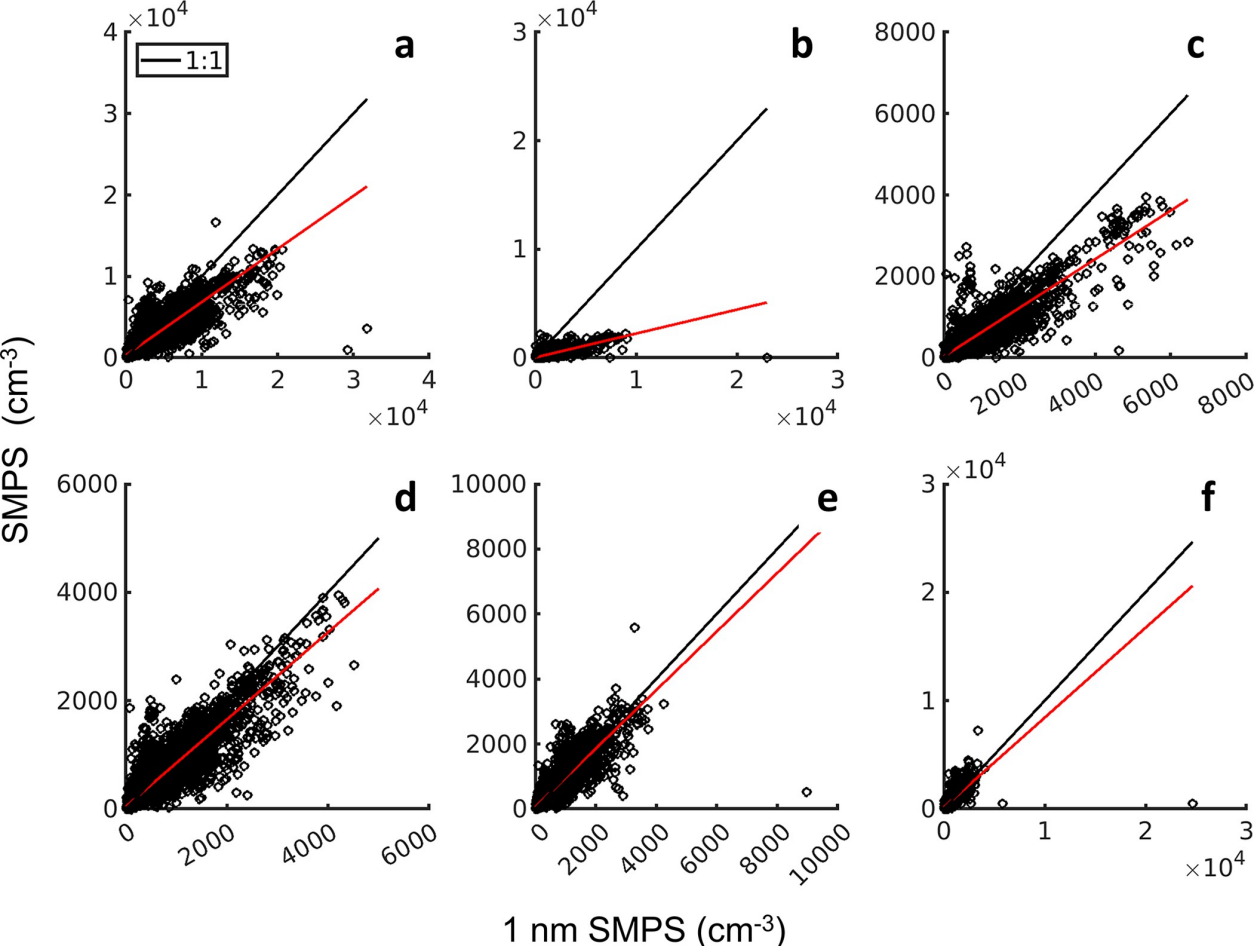
Scatter plots of the number concentrations in each bin of the overlap regions between the two SMPS instruments (a) 12–32 nm, (b) 12–16 nm, (c) 16–20 nm, (d) 20–24 nm, (e) 24–28 nm, and (f) 28–32 nm. Black lines are 1:1 and red lines are linear regression where the slopes are (a) 0.652, (b), 0.221, (c) 0.597, (d) 0.805, (e) 0.901, and (f) 0.830.

The 1 nm SMPS and standard SMPS overlapped in the 12–32 nm size range. Examining this overlap is important for quality assurance and for quantifying agreement between the two instruments. As the 1 nm SMPS had not been field deployed previously, and the site personnel were not familiar with it, checks against independent instrumentation are particularly important. The overlap region was divided into five size ranges and correlation between the two instruments was examined within each size range. The entire overlap region had a correlation coefficient (r) of 0.90 (Fig 6A). The most correlated size range was 20–24 nm (r = 0.93, Fig 6D). The least correlated size range was at the small end of the overlap region, 12–16 nm, with r of 0.82 (Fig 6B). Agreement (low mean difference) was best at the large sizes of the overlap. The mean response diverged with decreasing size, to about a 5:1 difference in the 12–16 nm size range. In other words, under 20 nm the 1 nm SMPS consistently measured higher number concentrations.

Several factors could contribute to this difference between the instruments. These include (a) the 1-nm SMPS was not dried while the SMPS aerosol and sheath air was dried; (b) the instruments had different inlets, separated by about 4 meters, and at different elevations relative to the ground; (c) the transmission efficiencies used in data processing are uncertain; (d) the difference in internal design and rod charge between the 1 nm and long DMA; and (e) the aerosol-to-sheath flow ratio influences the transmission resolution. It is important to note that 12–16 nm is near the lower limit of the SMPS and sizing and losses were not verified experimentally. The model 3086 1 nm DMA internal design has been optimized to reduce diffusion losses for particles <20 nm and increase the aerosol flow, and has a positively charged center rod while the long DMA is a negatively charged rod [48]. Intercomparison of the total (neutral plus charged) size distribution is contingent on the charging assumptions used in inversions (TSI inversion was used for both the 1-nm SMPS and the SMPS). The SMPS had an aerosol-to-sheath flow ratio of 1:4 compared to the 1 nm SMPS ratio of 1:10. However, the resolution difference is unlikely to cause the discrepancy in counting in the 12–20 nm size range. The lack of agreement in the 12–16 nm size range and the myriad of unresolved potential reasons for the discrepancy highlights the need for instrument intercomparison of sizing and counting instruments, especially those designed for nuclei mode particles.

### New particle formation / ultrafine burst events

**Fig 7:**
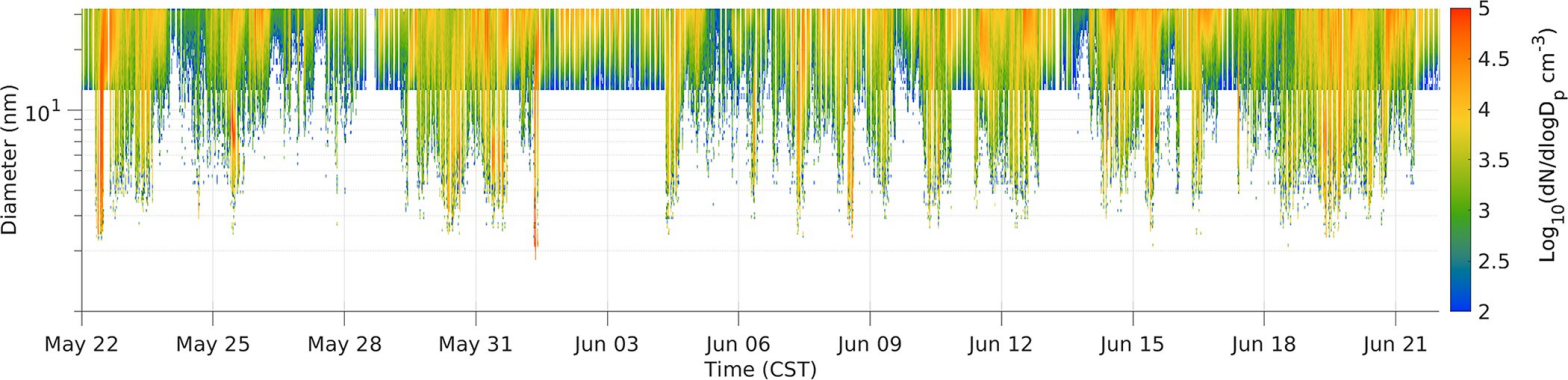
Number distribution from 1 to 30 nm at Zion. Individual days with UFP burst activity can be viewed in S1 File.

Particles in the 1–30 nm size range were highly variable in time (temporal evolution of the 1–10 nm size distribution is plotted in Fig 7). The average concentration in 1–3 nm size range (N_1-3_) was 18,000 cm^-3^, and the average concentration in the 3–10 nm size range (N_3-10_) was 1108 cm^-3^. These are much higher than the median concentrations, as shown in Fig 2, where the mean and median number PSD diverge starting at about 20 nm, and the median size distribution was zero below 5 nm.

Particles in the 3–10 nm size range (N_3-10_), occurred at lower concentration than the 10-month mean recorded in central Illinois by Bullard et al. [11] (1755 cm^-3^ in central IL vs. 1108 cm^-3^ for this study). Concentrations in this size range at continental locations have been observed to peak in spring and fall months, driven by H_2_SO_4_ and organic new particle formation and growth events [11, 12, 49]. The lower concentration of N_3-10_ at Zion vs. the central Illinois measurement is consistent with difference between the two studies in SO_2_ mixing ratio, taken as a proxy for sulfuric acid. Zion SO_2_ average was 0.32 ppb while the central Illinois mixing ratio was 0.87 ppb. Mean surface area concentrations were also higher at Zion (139.2 μm^2^ cm^-3^) than in Illinois (105.9 μm^2^ cm^-3^). The median and mean condensational sink for particle diameters 1.22–509.8 nm were 2.6 x 10^−3^ s^-1^ and .3.3 x 10^−3^ s^-1^, respectively. This is consistent with suppression of NPF at Zion via condensational scavenging to the Aitken mode of low volatility compounds responsible for nuclei formation and/or growth. It should be noted that month-to-month variability [11, 12] and measurement uncertainty in N_3-10_ are large, and only tentative conclusions can be drawn about the long-term average activity of new particle formation activity at Zion from the short measurement record of LMOS 2017.

Sources for comparison of the 1–3 nm time series and size distribution are limited. To the best of the authors’ knowledge, no other atmospheric size-resolved aerosol measurements have been conducted around the Great Lakes in the sub 5 nm size range for comparison. The first atmospheric measurements were collected during the 2011 summer in Atlanta, GA with a similar diethylene (DEG) SMPS configuration [50]. Their results show that the heights of the DEG SMPS size distributions function, dN/dlog(D_p_), are capable of reaching 1x10^7^ cm^-3^ during new particle formation events. Values reported for the PEGASOS 2012 campaign in Italy’s Po Valley were somewhat lower than for Zion in the 1.5–1.8 nm and 1.8–3 nm size bins, 2140 and 7980 cm^-3^ respectively; they further reported that the majority of the clusters were electrically neutral [51]. Thus the totals were 10,120 cm^-3^ for N_1.5–3_ in PEGASOS vs. 18,000 cm^-3^ for N_1.5–3_ during LMOS 2017. It should be noted that the PEGASOS campaign employed different instrumentation for detection. Kangasluoma et al. [52] presents an overview of current instrumentation for sub 10 nm particle measurements and concludes that measurements are still highly uncertain in this range and additional research is needed to improve the accuracy of these measurements.

Fourteen UFP burst events were identified qualitatively by the presence of rapid appearance of enhanced particles below 10 nm. More events would likely be identified if the 1 nm SMPS had fewer data gaps. In a few instances, these burst events were followed by continuous growth to larger (25–100 nm) sizes. More frequently, they were followed by disappearance of the nuclei mode or uneven and difficult to interpret temporal evolution of the size distribution [7, 53]. Thus conventional NPF growth rates were not calculated. All events began in the morning between 8:00 am– 1:00 pm CST. A table outlining the burst events (S3 Table in S1 File) and daily PSD plots have been included in the S1 File. The frequency of burst events is similar to that measured at Gunsch et al. [39] in Northern Michigan. There, 30% of days had NPF and growth activity, and the condensable compounds were due to a variety of sources, including long-range transport of urban and wildfire plumes, and to local biogenic emissions.

### Lake breeze and spray aerosol

During LMOS 2017, an onshore lake breeze occurred during numerous late mornings periods [33]. Averaging over six lake breeze days, a rapid and statistically significant increase in UFP concentration was seen, with a mode of 38 nm. N_20-80_ increased by about 4000 cm^-3^, from 8,410 cm^-3^ prior to lake breeze, to 12,440 cm^-3^ after lake breeze arrival. The position of the mode was identical to that reported in the only field observation of UFP LSA, that of Slade et al. [32], hereafter referred to as S11. During LMOS 2017, UFP increases occurred suddenly with lake breeze arrival in contrast with the slower (timescale of hours) observed buildup of secondary species (i.e., ozone and PM_2.5_) [34, 36, 54]. These similarities motivated our attempt to observationally confirm UFP from LSA, corroborate S11, and quantify the impact of LSA on UFP particles.

We analyzed the LMOS dataset in conjunction with buoy-measured wave heights to try to apportion UFP during onshore winds to three potential causal mechanisms. These are (a) UFP lake spray aerosol, (b) recent NPF and growth favored by chemical and meteorological features over the lake, and (c) primary particles emitted from nearby onshore sources at night, and then advected onshore in the lake breeze. Size-resolved chemical measurements at high time resolution [55] would likely be able to discriminate between these competing explanations; however, these require specialized instrumentation (absent from LMOS 2017) for particle mass spectroscopy at very small particle sizes. In the absence of size-resolved chemical measurements, we use the measured aerosol microphysical properties combined with records of wind speed, wind direction, and wave height to try to inform on the relative likelihood of the different possibilities. We also draw on previously reported LSA aerosol studies and discuss consistency of results.

**Fig 8:**
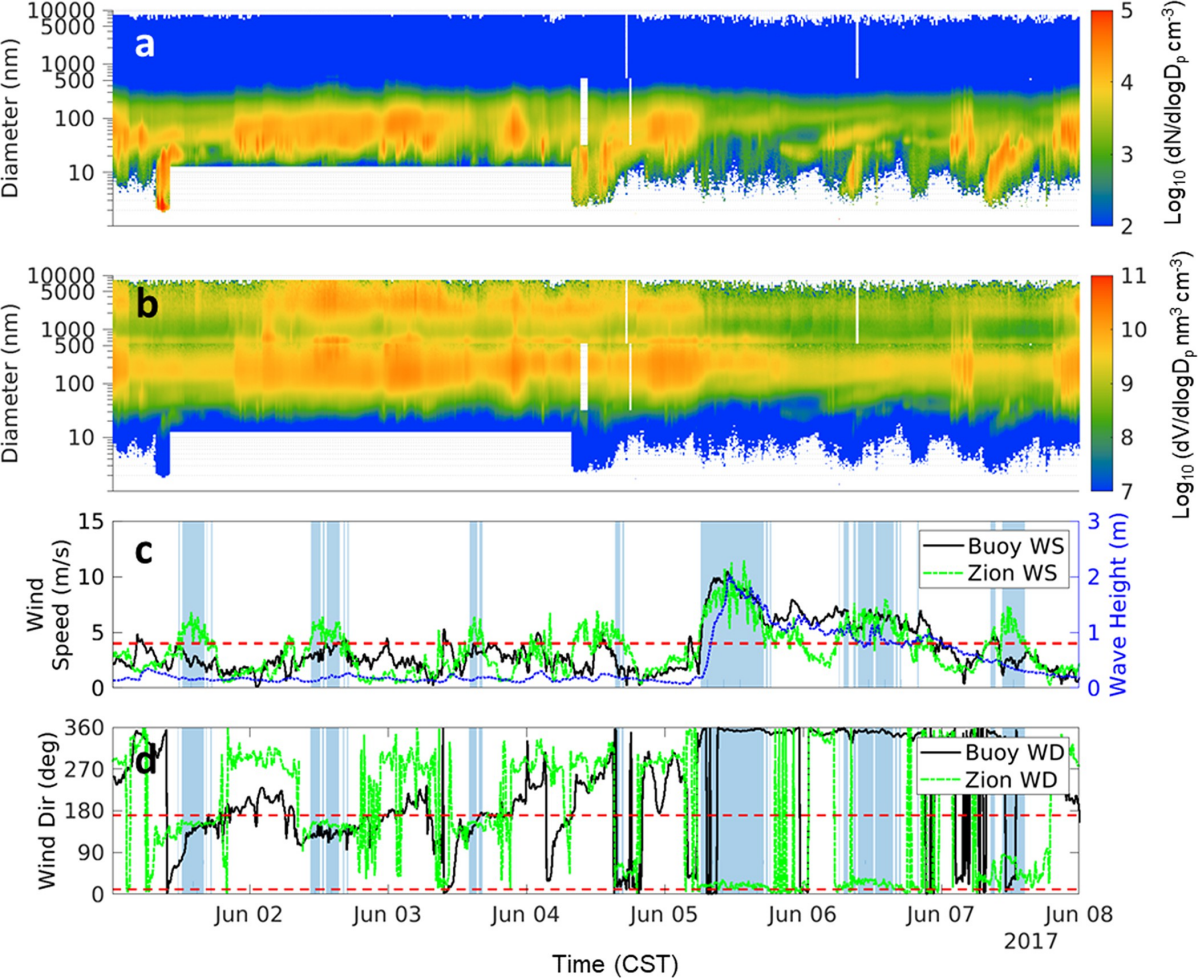
June 1- June 7 of 10 min PSD (a) and VSD (b); buoy wind speed (black), Zion wind speed (green), and wave height (blue) (c); buoy (black) and Zion wind direction (green) (d). Blue shaded region represents where Zion wind speed > 4 m/s and Zion wind direction between 10° and 170°.

The combined record of winds, wave height, and particle size distributions was inspected to check for evidence of UFP LSA. A data filter was implemented to identify times with the highest likelihood of LSA identification. This used onshore wind directions (10° to 170°) and high wind speeds (> 4 m/s) and is represented by the blue shading in Fig 8. We refer to these by the acronym Probable Detect of LSA (PDLSA) periods. Not used in the filter, but shown as an independent variable in Fig 8 is buoy-measured wave height at Wilmette Buoy, IL. The PDLSA periods often began after 10:00 am CST with the exception of June 5, 6, 7, and 14 where they began before 8:00 am CST and were sustained for several hours on average. The rationale of the PDLSA period is that if lake breeze is a strong source of UFP and particle number, then concentrations would increase at the onset of the PDLSA and decrease at the end of the PDLSA. Furthermore, within the PDLSA periods, observed variation in wind speed would correlate positively with particle number, and possibly with mode shifts.

During the PDLSA periods, wave heights above 1 m occurred on June 5, 6, and 20; therefore, these are the best cases for confirming and/or quantifying an LSA source of UFP. In all three cases, onshore wind direction were from the northeast. None of the cases are consistent with a strong UFP LSA source. On June 5, number concentrations decreased at PDLSA onset, and remained approximately constant at the end of the PDLSA period. On June 6, number concentrations were unaffected by onset or end of the PDLSA period. And on June 20, particle number dropped at PDLSA onset and then increased at the end of the PDLSA.

**Fig 9:**
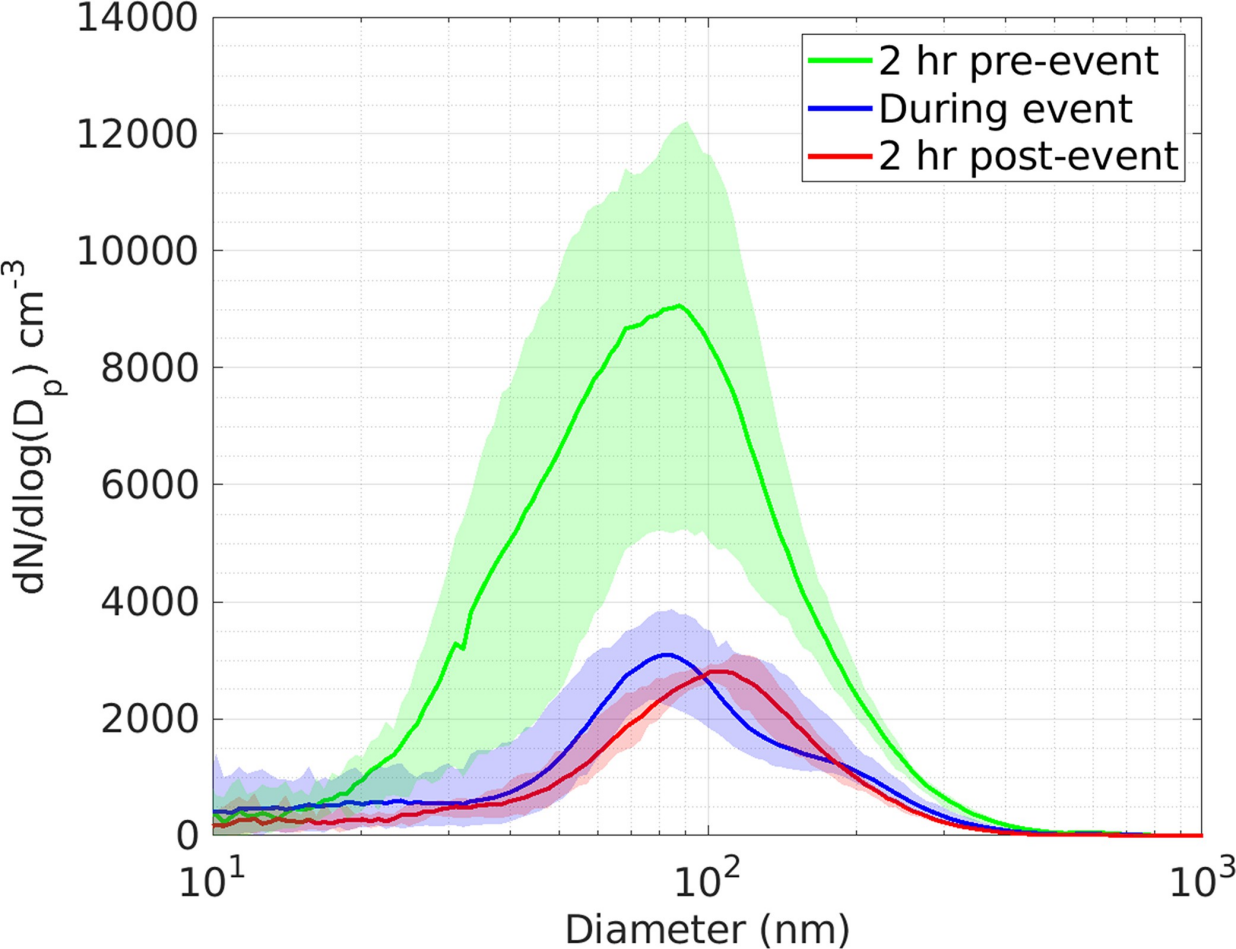
Arithmetic mean of PSD on June 5 for 2 hours prior (green), during (blue), and 2 hours post (red) identified wave breaking conditions. The shaded regions represent 5^th^– 95^th^ percentiles.

The June 5 event was selected as an ideal candidate to investigate LSA influence on the UFP portion of the PSD. Wind direction was consistently from the north to northeast which likely reduces the influence of urban air plumes from the southern coast of the lake. Wave height above 1 m was sustained for several hours and peaked at 2 m, the highest recorded height during the campaign. This was the longest PDLSA period during the campaign lasting from 6:00 am to 5:00 pm CST. In Fig 9, we show the PSD function averaged for 2 hours prior to PDLSA onset (green), during the PDLSA period (blue), and for the 2 hours immediately following the PDLSA period (red). The height of the PSD function (dN/dlogD_p_) from 9000 cm^-3^ prior to the event to 3000 cm^-3^ during and after the event and similar trend is observed in the volume distributions (not shown). While the number distribution mode of 81 nm was is in agreement with the first mode (80 nm) of the synthetic freshwater LSA reported in May et al. [23] and Harb et al. [56], the change in concentration is in the wrong direction to support a strong UFP LSA signature. Furthermore, the mode position and size are inconsistent with those in S11, where the mode was at smaller particle diameters (20–40 nm) and more intense (reaching 14,000 cm^-3^), implying an number enhancement of over 3000 cm^-3^ to particle count during winds of 6.5 m/s. In other words, corroboration of S11 would require a much more intense “During event” trace from LMOS 2017.

In a third effort to confirm existence and quantify strength of an LSA UFP contribution, we subjected all 2-min data of wind speed and particle number with onshore flows and wind speeds in excess of 2 m/s, to regression analysis. The estimated autocorrelation was 0.99. UFP particle number showed a negative association with wind speed, but the association lacked statistical significance (p = 0.24).

In conclusion we were unable to show existence of an LSA impact on UFP particle number or size distribution. An effect may exist; however, if it does, it is likely much smaller than that reported in S11. Furthermore, it is difficult to isolate given other sources of variability, and single particle mass spectroscopy and/or elemental analysis of single particles via transmission electron microscopy would be needed. Furthermore, locating samplers on the lake (ship-borne) or directly on the shore or on a pier, and at locations with minimal anthropogenic influence would increase signal-to-noise ratio.

We furthermore show that LSA UFP in S11 is likely a false positive detection. S11 reported results of aircraft observations over northern Lake Michigan taken in 2009. Correlation was noted between wind speed and particle number in the 15–40 nm size range at the lowest flight elevations. Breaking waves were observed during the higher wind speeds. The magnitude of the enhancement was 100–400 cm^-3^ at wind speeds below 5.5 m/s. Two high leverage data points, corresponding to flights on July 16, 2009, and August 2, 2009, exhibited particle enhancement of 1000–3000 cm^-3^; these cemented the strong positive relationship between wind speed and particle number. However, this was a small sample size, with seven total flights–and no chemical confirmation that the UFP where chemically similar to concentrated lake spray. In an NPF and growth study concurrent with the flights used in S11, Kanawade et al. [12] measured the PSD, SO_2_, sulfuric acid, isoprene, and other biogenic VOC, at a forested site 25 km from the shore of Lake Michigan. They reported a surprising absence of midday NPF events, and attributed it to the clean conditions (lack of SO_2_ and NH_3_) and to isoprene suppression of OH and NPF. However, they reported two (and only two) NPF events. However, the two events occurred precisely on the two days (July 16 and August 2) with the highest particle numbers observed in S11. In agreement with S11, the events produced narrow PSD with modes ranging from about 10 to 50 nm. However, Kanawade et al. (2012), using chemical tracers and back trajectory analysis, attributed the particles to SO2-driven NPF activity. In other words, the two data points that drove S11 to conclude UFP are from LSA are entirely consistent with regional NPF and growth. Finally, S11 contained an additional observed detail that is better reconciled by NPF as the source of the mode than LSA. On August 2, 2009, N_15-40_ was measured at low altitude (130 m) and at higher altitude (430 m). Contrary to expectation from an LSA source, the concentration was low near the lake surface, and higher at elevation. Such behavior is common in NPF and growth events that occur in stratified conditions or in convective updrafts [57].

Given the combined evidence, of the three potential causes of enhanced UFP in onshore breezes, NPF activity is the cause most consistent with data. Its detection during LMOS, its widespread detection in the region [12, 39, 57] including on high wind speed days with northerly winds, the availability of precursors to nucleation and growth, and the lack of relationships with UFP and wind speed demonstrated in this work, point to NPF as the key driver of the number enhancement. High LSA impacts such as 1000–3000 cm^-3^ UFP under 2 m breaking waves as proposed in S11 are simply inconsistent with our LMOS 2017 measurement record.

We note that the lower impacts simulated in WRF-Chem by Chung et al. [30] for LSA number during for summer 2004 may be consistent with the observational record. The model attributed about 150 cm^-3^ of UFP to LSA over southern Lake Michigan, and about 100 cm^-3^ at coastal locations such as Zion. Peak periods were associated with impacts about twice the average. It was noted that LSA inhibited NPF activity in the model due to increases in condensational sink. And with stronger LSA emissions (or weaker NPF and growth activity), the inhibition effect can lead to zero net increase in particle number upon LSA generation. This perhaps generates a fourth hypothesis that warrants future study: UFP LSA generation occurs, but it inhibits NPF activity leading to small impacts on total number with high variability due to the specifics of oxidation chemistry and particle dynamics in each case.

Finally, we emphasize that this work does not contribute to the discussion or quantification of coarse mode LSA. Coarse mode LSA has been generated in laboratory studies and confirmed observationally in the field [27, 58, 59].

### Spatial context and air quality implications

During LMOS 2017, the PM_2.5_ concentration at Zion had mean values of 5.2 μg m^-3^ (filters) and 6.4 μg m^-3^ (reconstructed from PSD), with a standard deviation (at 2-min time resolution) of 4.3 μg m^-3^. These are likely representative of urban-influenced background sites of the Great Lakes. These compare favorably to current US air quality standards (12 μg m^-3^, annual PM_2.5_; 35 μg m^-3^ daily PM_2.5_), but are over the WHO guideline of 5 μg m^-3^. Spatial variability of PM_2.5_ was not resolved through observations during LMOS 2017 except through variation in particle concentration with the wind direction, and consideration of emissions from known sources [35]. We have shown through conditional probability analysis that particle concentrations of all sizes were, at Zion, more prevalent from sources to the west and in lake breezes originating to the southeast. Strong near-field PM_2.5_ influences were shown to be limited, thus supporting spatial homogeneity of PM at approximately 4 km spatial averaging.

**Fig 10:**
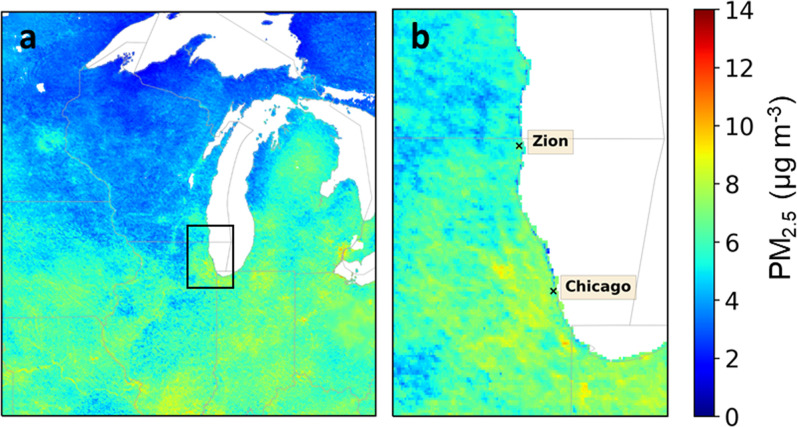
Estimated mean PM_2.5_ for June 2017 for upper US Midwest (a) and southwest shore of Lake Michigan (b) as determined by combining AOD satellite retrievals with GEOS-Chem and calibrated with ground observations using geographically weighted regression [41].

The estimated mean PM_2.5_ concentrations (spatial resolution of 1 km^2^) in Fig 10 highlights moderate variation domain wide of 2–11 μg m^-3^ with the higher concentrations in the urban areas around the southern Great Lakes (Lake Erie and Lake Michigan). This also supports the higher concentrations of PM_2.5_, ranging from 7–10 μg m^-3^ around the southwestern coast of Lake Michigan and the Zion average was estimated to be 6 μg m^-3^. PM_2.5_ is not estimated over the Great Lakes, precluding discussion of LSA or other aerosol sources over water.

NO_2_, on the other hand, was measured over both land and water with high spatial resolution during LMOS 2017. Sampling was done with GeoTASO at spatial resolution of 250 m x 250 m [60]. Furthermore, NO_x_ and PM_2.5_ were correlated at Zion with a slope of 0.61 μg m^-3^ per ppb of NO_x_ and a coefficient of determination (r^2^) of 0.51. Thus, pseudo-PM_2.5_ mapping may be feasible (or downscaling of existing modeled or satellite-model-surface observation fusion) may be possible using NO_2_ remote sensing. Collocated PM_2.5_ and NO_2_ are available at several sites in the region and could be used to further assess the viability of downscaling PM_2.5_ to detect hotspots over current or future standards. Several machine learning algorithms and regression modeling have supported that the relationship between PM_2.5_ and co-pollutants (NO_2_, SO_2_, and O_3_) are important variables to consider when estimating PM_2.5_ concentrations at high spatio-temporal resolutions and implications to epidemiological studies [61–63]. These methods are expected to continue to improve with the addition of geo-stationary satellites (TEMPO, GEMS) which will provide more temporally resolved data products.

## Summary and conclusions

During the LMOS 2017 field campaign, the PSD was measured at 2-min time resolution in the size range 1.02 nm to 8.671 μm. At this coastal Lake Michigan site, average conditions during the study were characteristic of clean rural or urban periphery sites in the region, with occasional urban and point source impacts. The site can be characterized by its averages in aerosol optical depth (0.084), reconstructed PM_2.5_ (6.4 μg m^-3^), reconstructed PM_10_ (7.9 μg m^-3^), SO_2_ (0.32 ppb), and mean number concentrations for the 1–3 nm and 3–8761 size ranges: 1.80x10^4^ cm^-3^ and 7998 cm^-3^, respectively.

Instrument intercomparisons were conducted and reported herein to show consistency across instruments for both particle number and particle mass. One instrument-to-instrument disagreement was that under 16 nm, the 1 nm SMPS did measure consistently higher number concentrations with a 5:1 ratio.

Only a handful of conventional new particle formation events (i.e., appearance of particles at nuclei sizes with smooth growth to larger sizes such as 25–100 nm) were detected during the campaign. However, bursts of ultrafine particles were observed on about half of study days, originating in the 3–10 nm size range.

A specific investigation of ultrafine LSA was conducted due to opportunistic occurrence of sustained wave breaking conditions and onshore flow. The relationship between particle number and wind speed had a negative slope which was not statistically significant (p = 0.24). The alternative hypothesis of enhanced UFP in onshore flow as originating from NPF and growth activity is more likely. Inhibition of NPF by LSA, as proposed in Chung et al. [30], may be masking a source of UFP particles during high wind / breaking wave conditions. Future confirmation efforts for UFP from LSA should focus on (a) more northerly sites with lower anthropogenic influence, (b) chemical composition and microscopy at the size ranges of interest, (c) vertical profiles from aircraft with simultaneous surface monitoring on shore, and (d) sites in located on or very close to the lake.

## Supporting information

S1 File Files for size resolved aerosol at a Coastal Great Lakes Site: Overview, Sub 3-nm sizing instrumentation results, and ultrafine lake spray aerosol. (DOCX)
