# Supplementary material for: Size-resolved aerosol at a Coastal Great Lakes Site: Impacts of new particle formation and lake spray
Source: PLoS One. 2024 Apr 4;19(4):e0300050. doi: 10.1371/journal.pone.0300050 (PMC10994298; doi:10.1371/journal.pone.0300050)
Supplement: S1 File — (DOCX) [file pone.0300050.s001.docx]

**Supplemental Information Files for Size Resolved Aerosol at a Coastal Great Lakes Site: Overview, Sub 3-nm Sizing Instrumentation Results, and Ultrafine Lake Spray Aerosol**

**S1 Table. A summary of local and NARSTO flags used on instrumental data for quality assurance during the campaign.**

| Local Flag | NARSTO Flag | Description |
| --- | --- | --- |
| DRY | M2 | Diffusion dryer and drierite tube change |
| FLO | M2 | Flow tests |
| DRN | M2 | CPC water drain |
| INL | M2 | Not sampling through inlet |
| HEP | M2 | Leak check with HEPA filter |
| RHP | M2 | RH probe check |
| STA | M2 | Sampling trailer air |
| NET | V6 | Neutralizers switched (x-ray and Kr-35) |
| TRB | M2 | Troubleshooting instrument |
| UNK | M2 | Unusual high counts |
| PUM | M2 | CO2 pump not functioning |
| CAL | M2 | SO2 span check with calibration gas |
| ZER | M2 | SO2 zero test with zero air generator |

**S2 Table. Statistics for particle variables measured during the campaign**

| Variable | Time Avg (min) | Units | N | Mean | Std Dev | Min | 5th | Median | 95th | Max |
| --- | --- | --- | --- | --- | --- | --- | --- | --- | --- | --- |
| PM_2.5_ | 2 | µg m^-3^ | 18575 | 6.4 | 4.0 | 1.1 | 1.9 | 5.2 | 14.1 | 23.6 |
| PM_10_ | 2 | µg m^-3^ | 18575 | 7.9 | 5.0 | 1.3 | 2.3 | 6.6 | 16.9 | 37.7 |
| CPC^†^ | 2 | cm^-3^ | 8787 | 6469 | 4,1107 | 753 | 1,317 | 5,838 | 13,914 | 42,753 |
| PN_p_(3 – 8671 nm)* | 2 | cm^-3^ | 8787 | 8,485 | 5,616 | 1,020 | 1,717 | 7,637 | 18,145 | 1.19 x10^5^ |
| PN (1 – 3 nm) | 2 | cm^-3^ | 14964 | 1.80x10^4^ | 1.64x10^5^ | 0 | 0 | 0 | 7,957 | 8.261 x10^6^ |
| PN (3 – 10 nm) | 2 | cm^-3^ | 14964 | 1,108 | 2,804 | 0 | 0 | 268 | 4,663 | 1.141 x10^5^ |
| PN (10 – 500 nm) | 2 | cm^-3^ | 13126 | 6,895 | 4,623 | 781 | 1,572 | 5,938 | 13,126 | 5.348 x10^4^ |
| PN (3 – 2168 nm) | 2 | cm^-3^ | 13126 | 7,993 | 5,919 | 783 | 1,608 | 6,835 | 1.79 x10^4^ | 1.191 x10^5^ |
| PN (3 – 8671nm) | 2 | cm^-3^ | 13126 | 7,998 | 5,921 | 783 | 1,608 | 6,836 | 1.79 x10^4^ | 1.191 x10^5^ |
| PS (1 – 3 nm) | 2 | µm^2^ cm^-3^ | 14964 | 0.089 | 0.784 | 0 | 0 | 0 | 0.065 | 41.23 |
| PS (3 – 500 nm) | 2 | µm^2^ cm^-3^ | 19519 | 139.2 | 87.3 | 12.8 | 43.1 | 111.0 | 301.6 | 608.6 |
| PS (3 – 2168 nm) | 2 | µm^2^ cm^-3^ | 18575 | 147.9 | 92.2 | 14.8 | 44.8 | 119.2 | 319.9 | 621.9 |
| PV (3 - 2168 nm) | 2 | µm^3^ cm^-3^ | 18575 | 4.78 | 2.99 | 0.83 | 1.44 | 3.90 | 10.60 | 17.73 |
| PV (3 – 8671nm) | 2 | µm^3^ cm^-3^ | 18575 | 5.97 | 3.74 | 0.95 | 1.76 | 4.94 | 12.72 | 28.37 |
| AOD_550_ | NA | unitless | 404 | 0.084 | 0.0511 | 0.021 | 0.026 | 0.074 | 0.183 | 0.249 |

* statistics for June 1 – 21, 2017 for more direct comparison to CPC concentrations.

† statistics for the corrected CPC.

PN = Particle number, PS = Particle Surface Area, PV = Particle Volume

**S3 Table. NPF/UFP burst event dates and times.**

| Date | Start Time (CST) | End Time (CST) | comment |
| --- | --- | --- | --- |
| 5/22 | 9:00 | 19:00 | Growth of particles did not exceed 50 nm. |
| 5/23 | 9:00 | 15:00 |  |
| 5/25 |  |  | significant particles in sub 10 nm range from 9:30 to 1:00 pm. Possible growth starting at 20 nm at 11:00 am, appears interrupted or discontinuous |
| 5/30 | 9:00 | 13:00 | potential growth pattern interrupted at 1:00 pm. |
| 6/1 | 8:00 | 12:00 | high particle numbers below 3 nm 8:30 – 9:30 am. |
| 6/4 | 11:00 | 6:00 (6/5) |  |
| 6/6 | 8:00 | 10:00 | doesn't seem to start in nucleation range, smaller event from 14 to 18 UTC, but looks to have never left nucleation range |
| 6/7 | 7:30 | 16:00 | somewhat linear growth that never exceeded ~60 nm |
| 6/8 | 12:00 | 15:00 | sporadic ultrafine particles between 3 and 15 nm |
| 6/10 | 8:00 | 12:00 |  |
| 6/12 | 8:00 | 11:00 |  |
| 6/15 | 9:30 | 23:00 |  |
| 6/16 | 11:00 | 3:00 (6/17) |  |
| 6/19 | 16:00 | 19:30 |  |


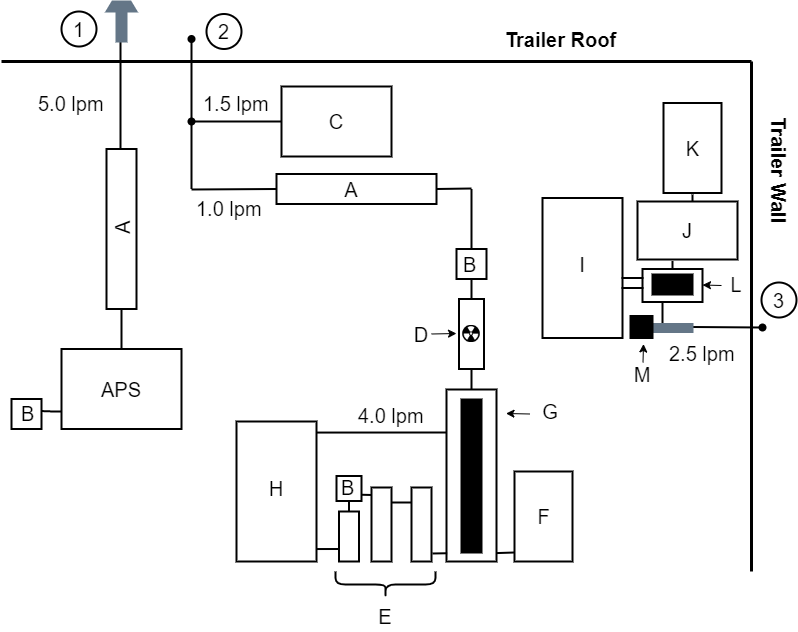


S1 Fig. Aerosol instruments deployed during LMOS. Numbers refer to inlets: APS equipped with the PM_10_ inlet (1), SMPS and CPC inlet (2) and 1 nm SMPS inlet (3) consisting of bug and rain guards. Letters refer to: diffusion dryers (A), RH sensors (B), butanol CPC 3025 (C), Kr-85 neutralizer (D), drierite tubes with HEPA filter (E), water CPC 3785 (F), long DMA 3081 (G), classifier 3080 (H), classifier 3082 (I), DEG nano enhancer 3777 (J), butanol CPC 3772 (K), 1 nm DMA 3086 (L), and soft x-ray neutralizer (M).


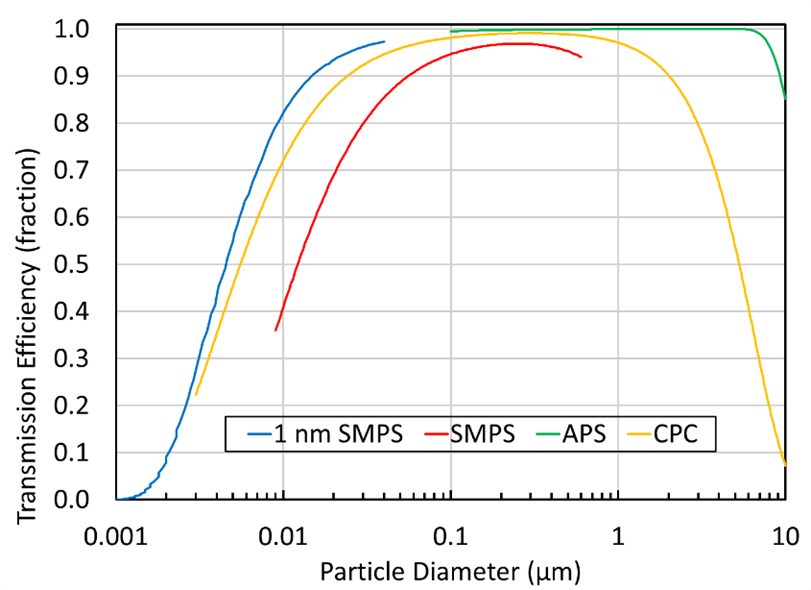


**S2 Fig. Aerosol transmission efficiency curves, corrected for sampling inlets, for the APS (green), CPC(yellow), SMPS (red), and 1 nm SMPS (blue).** The 1 nm SMPS curve includes neutralizer and instrumental losses.


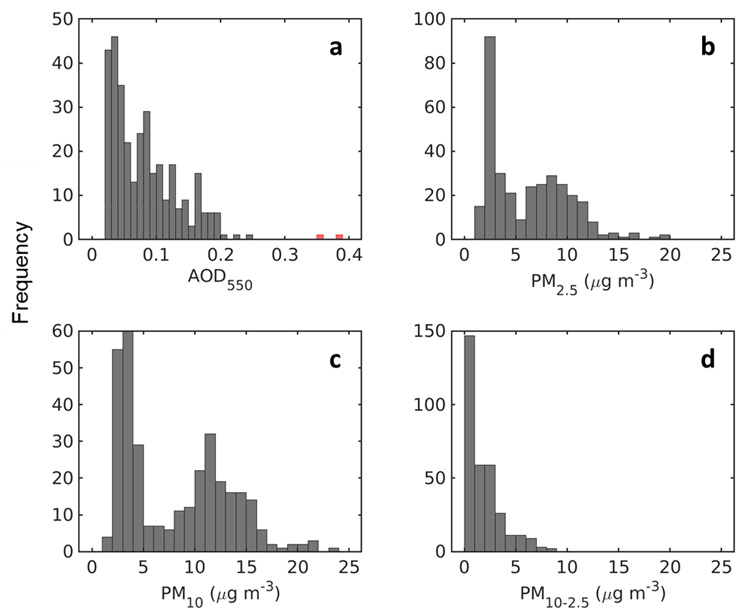


**S3 Fig. AOD_550_ (a), PM_2.5_ (b), PM_10_ (c), and PM_coarse_ (d) (histograms) where the PM measurements are the 2 min averages that coincide with each AOD measurement.** The red bars in (a) coincide with observations on June 13 that were excluded.


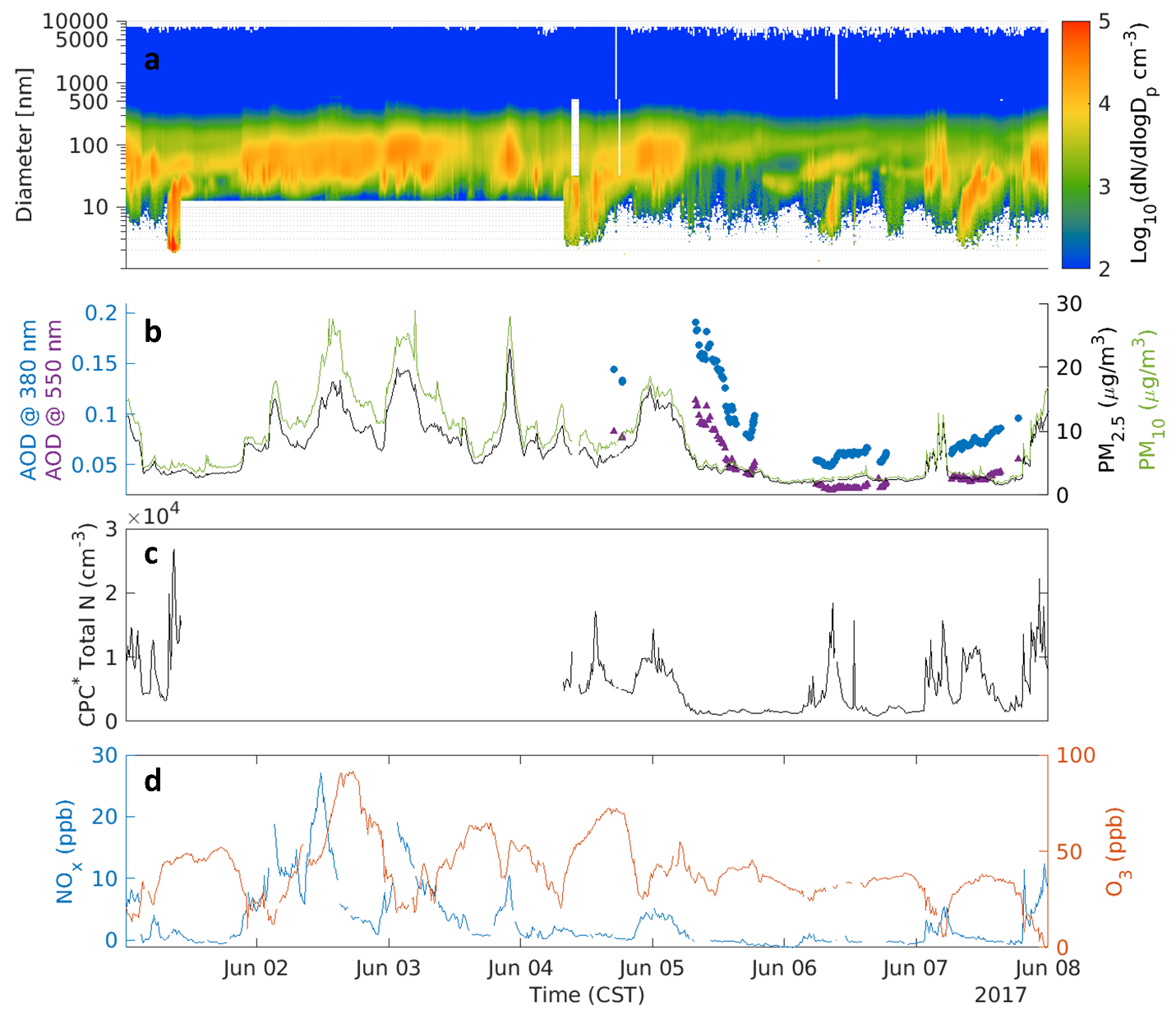


**S4 Fig. Selected gas phase and aerosol variables for June 1 – 7, 2017 averaged to 10 min (except AOD).** Timeseries of the particle size distribution in panel a, AOD_380_ (blue dot), AOD_550_ (purple dot), PM_2.5_ (black), and PM_10_ (green) in panel b, total CPC number concentration in panel c, and NO_x_ (blue) and ozone (orange) in panel d.

**
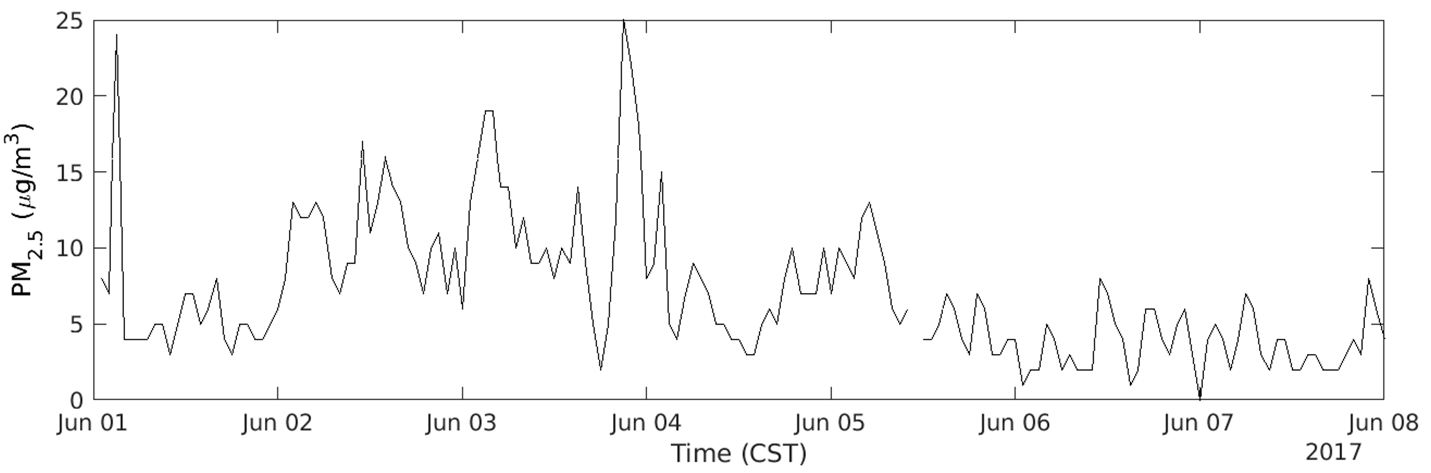
**

**S5 Fig. Hourly PM_2.5_ from Chiwaukee Prairie from June 1 – 7, 2017.**

**
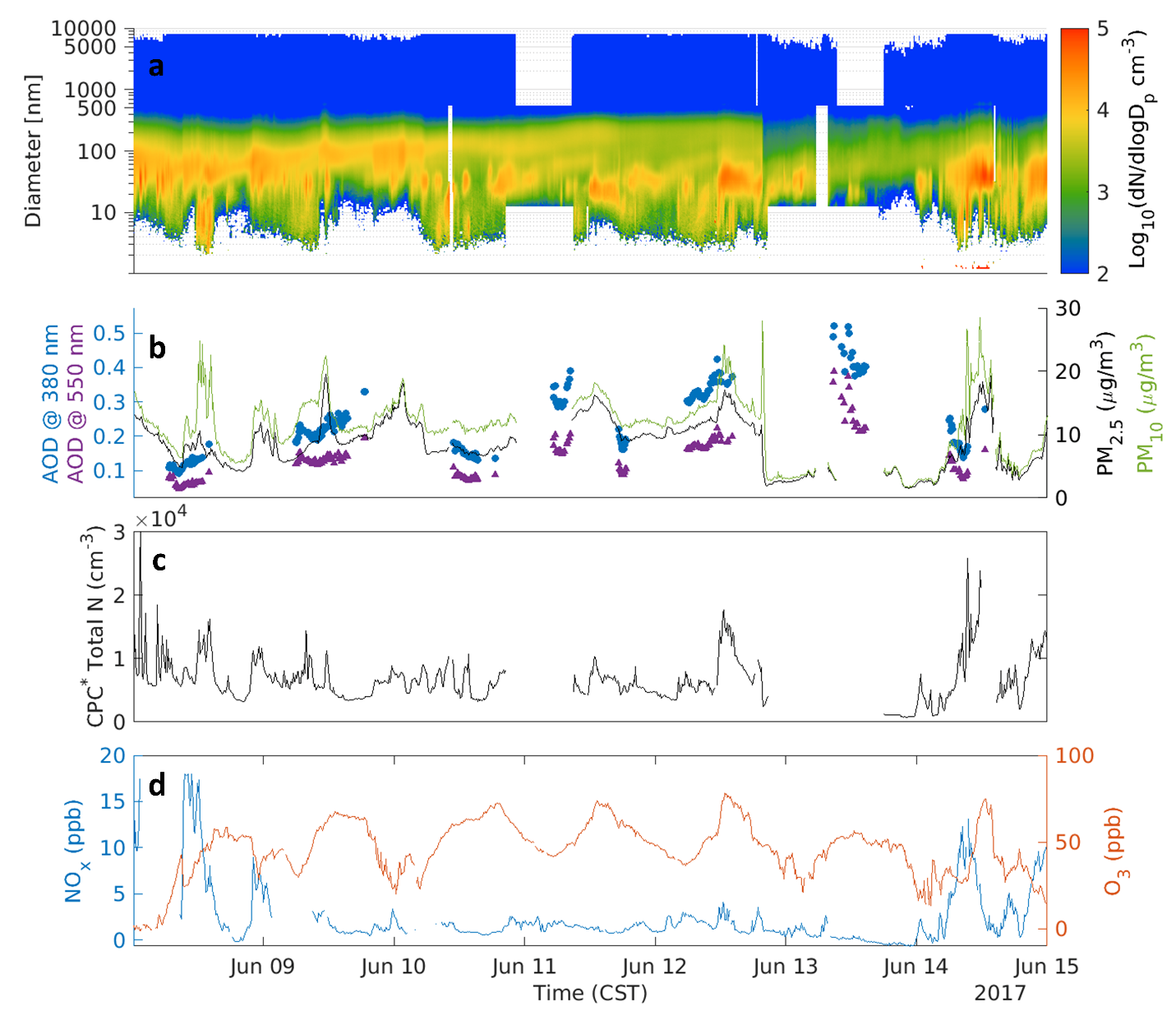
**

**S6 Fig. Same as S4 Fig for June 8 – 15, 2017.**

**
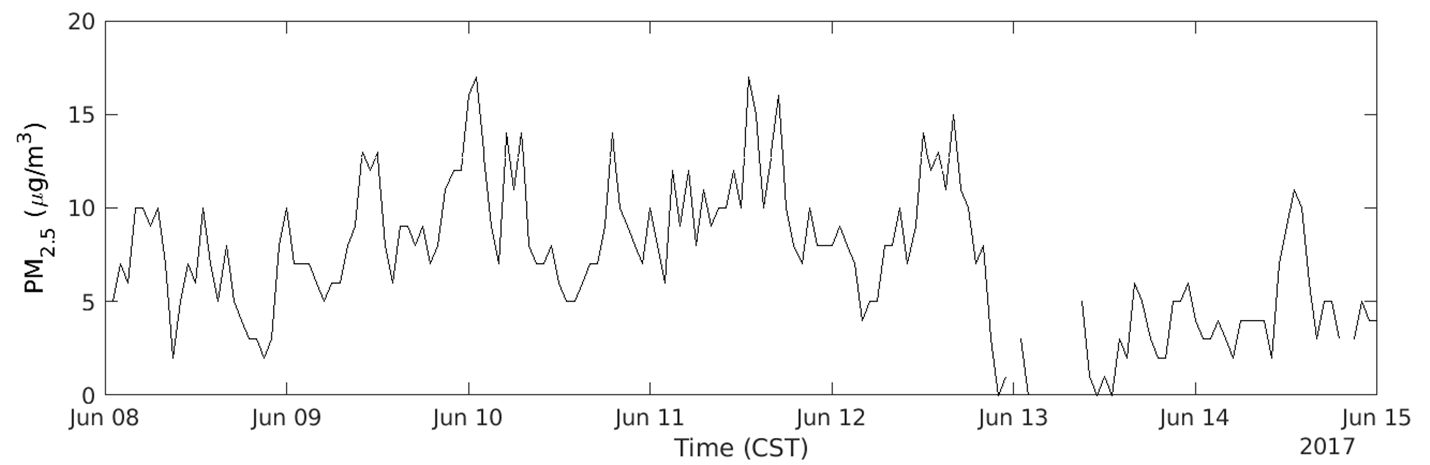
**

**S7 Fig. Hourly PM_2.5_ from Chiwaukee Prairie from June 8 – 15, 2017.**

**
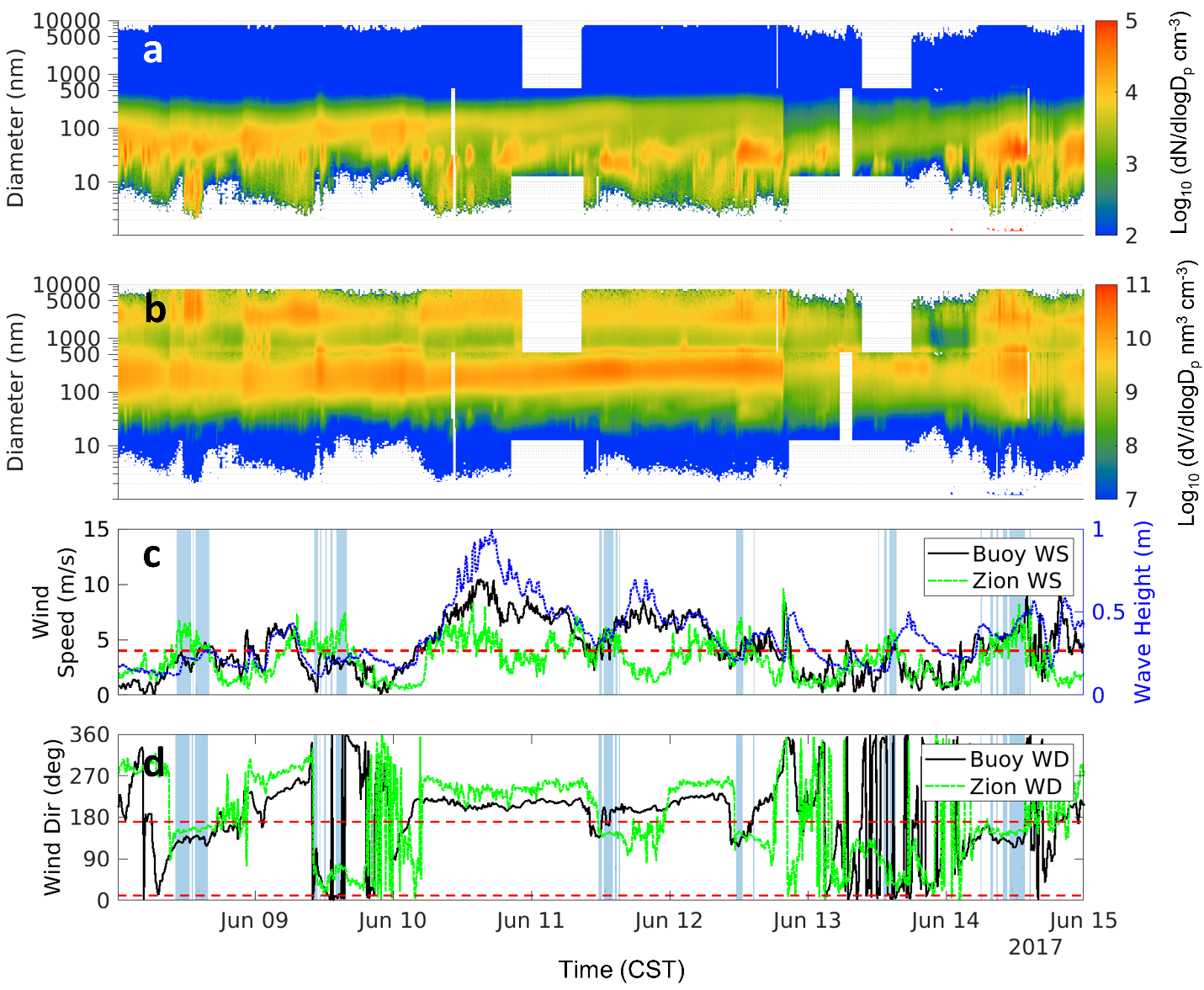
**

**S8 Fig. June 8 – 15, 2017 of 10 min PSD (a) and VSD (b); buoy windspeed (black), Zion windspeed (green), and wave height (blue) (c); buoy (black) and Zion wind direction (green) (d).** Blue shaded region represents where Zion windspeed > 4 m/s and Zion wind direction between 10° and 170°.

**
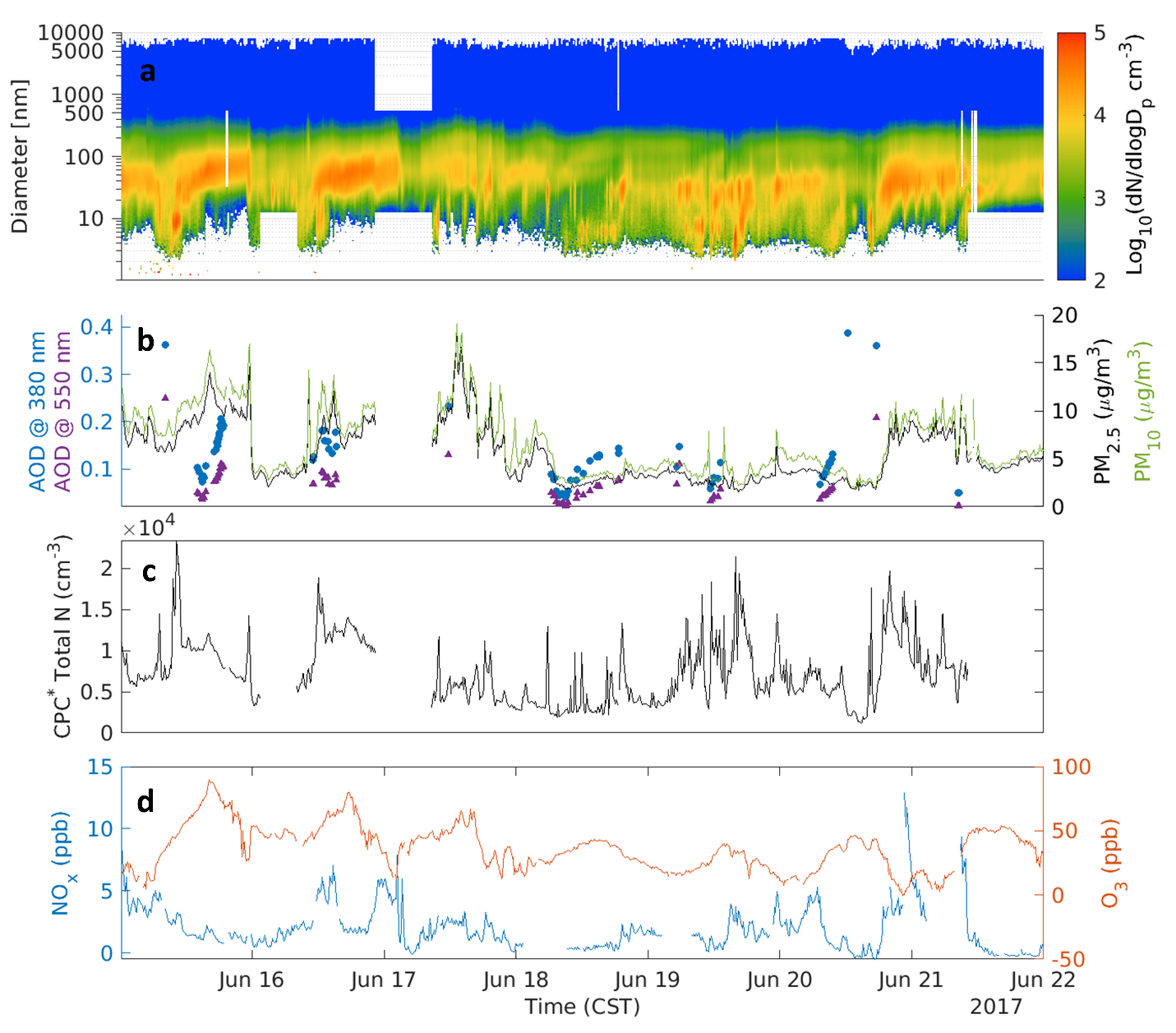
**

**S9 Fig. Same as S4 Fig for June 15 – 22, 2017.**

**
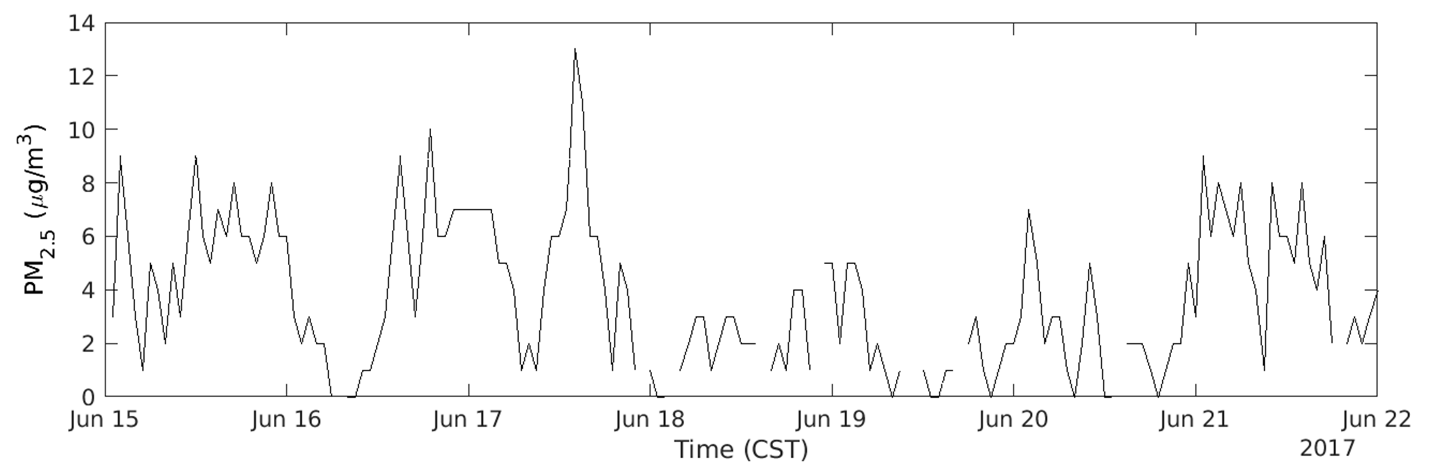
**

**S10 Fig. Hourly PM_2.5_ from Chiwaukee Prairie from June 15 – 22, 2017.**

**
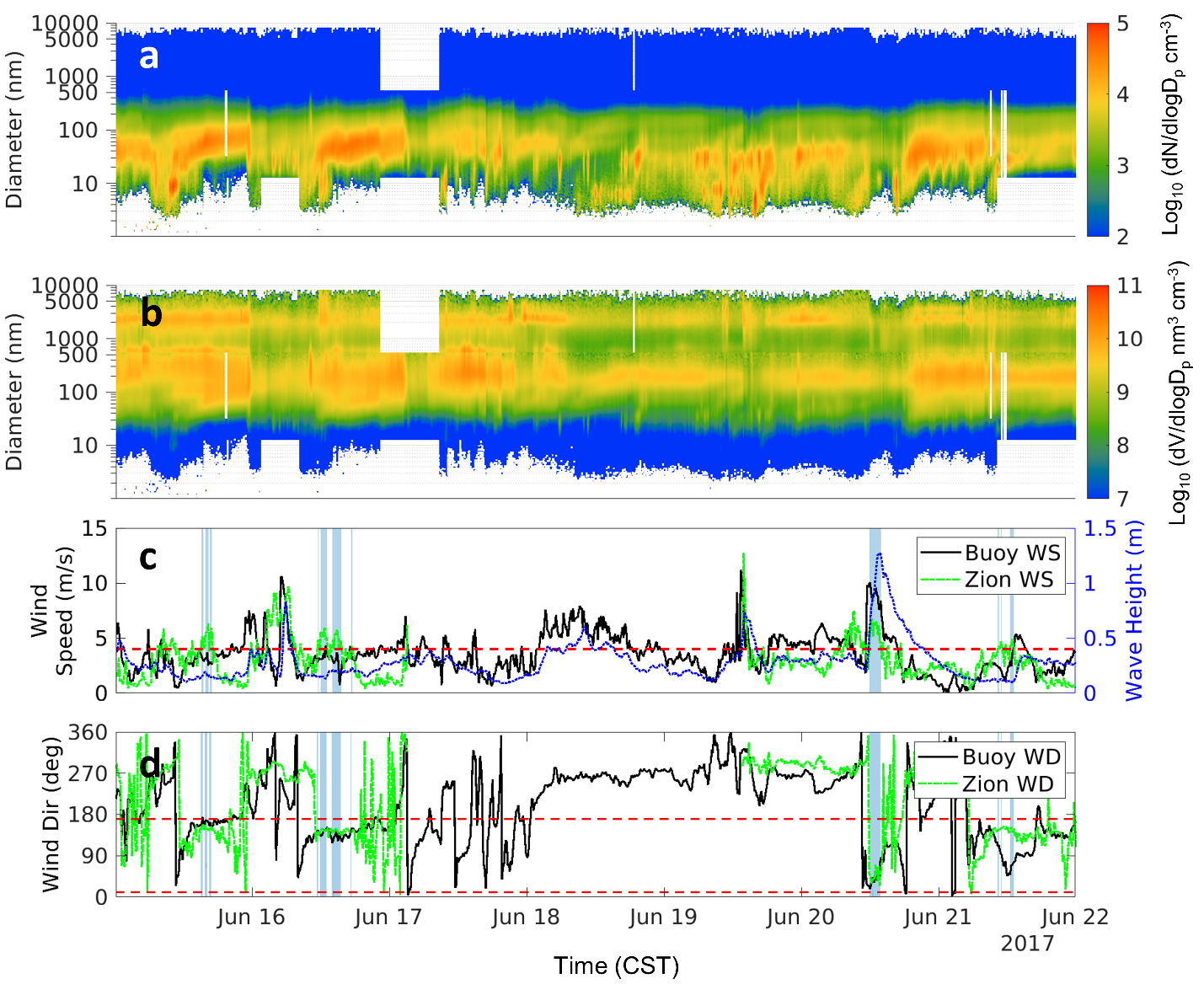
**

**S11 Fig. The same as S8 Fig for June 15 – 22, 2017.**


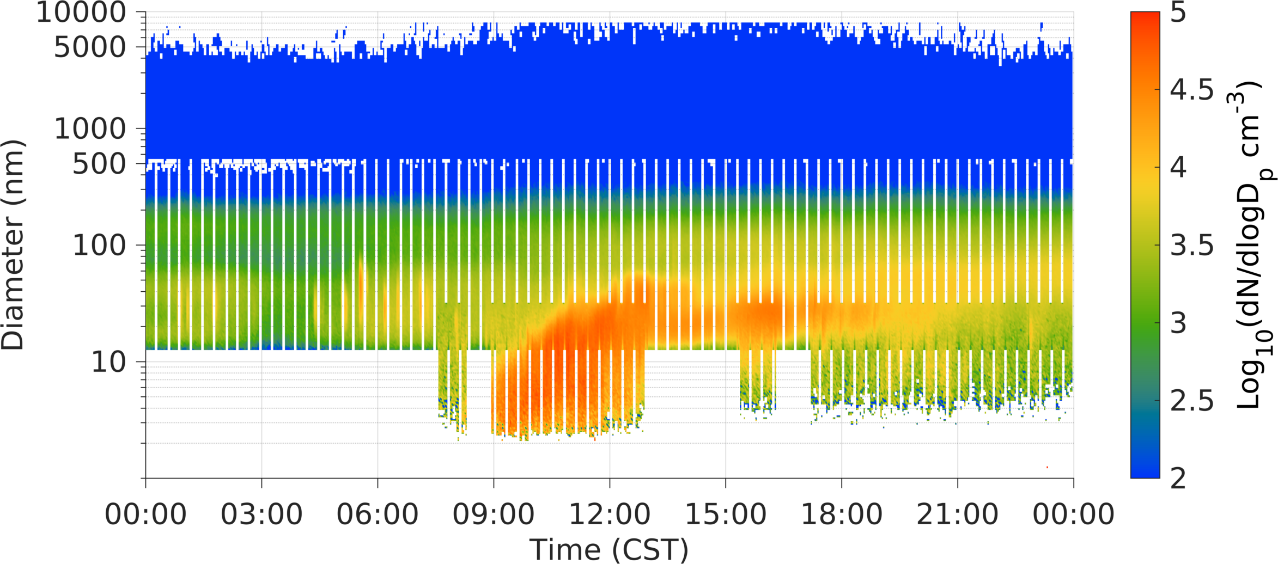


**S12 Fig. May 22, 2017 of 2 min particle size distribution.**

**
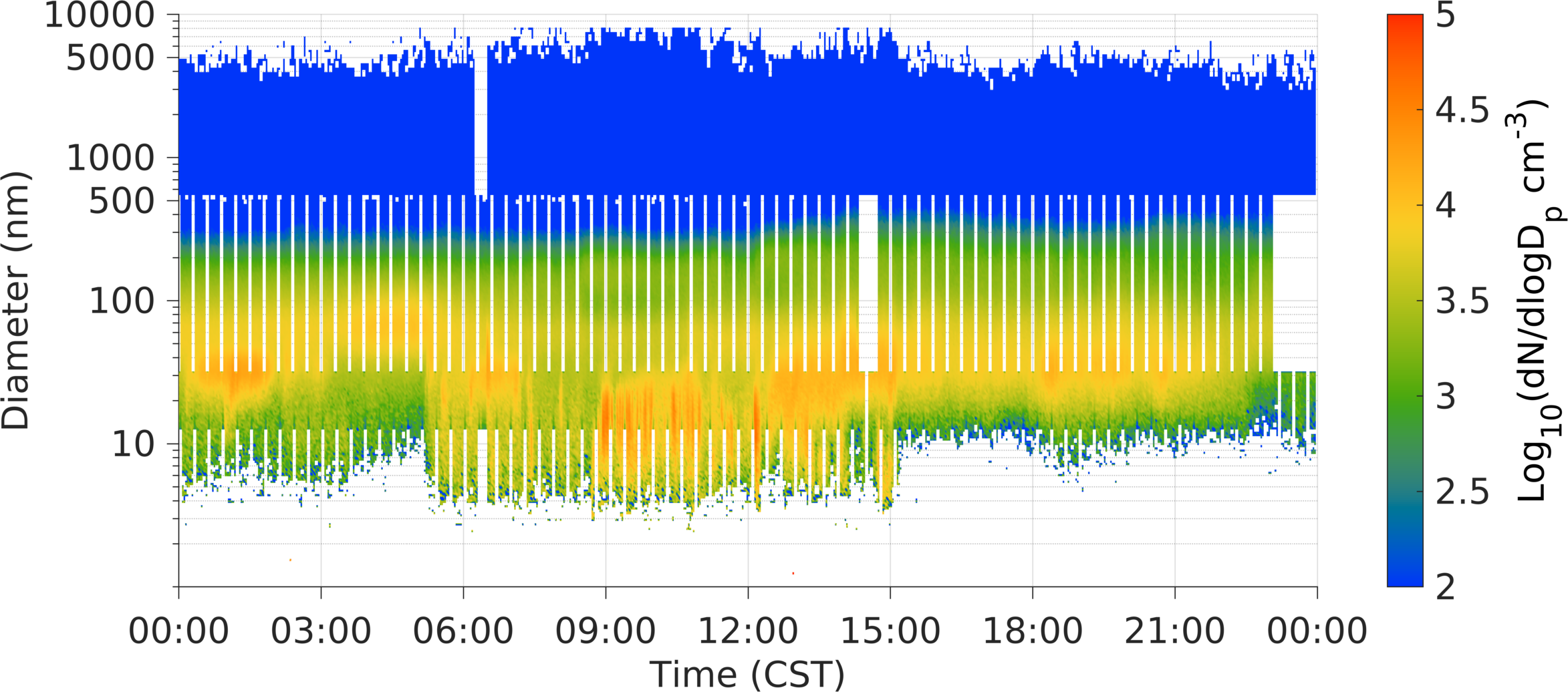
**

**S13 Fig. May 23, 2017 of 2 min particle size distribution.**

**
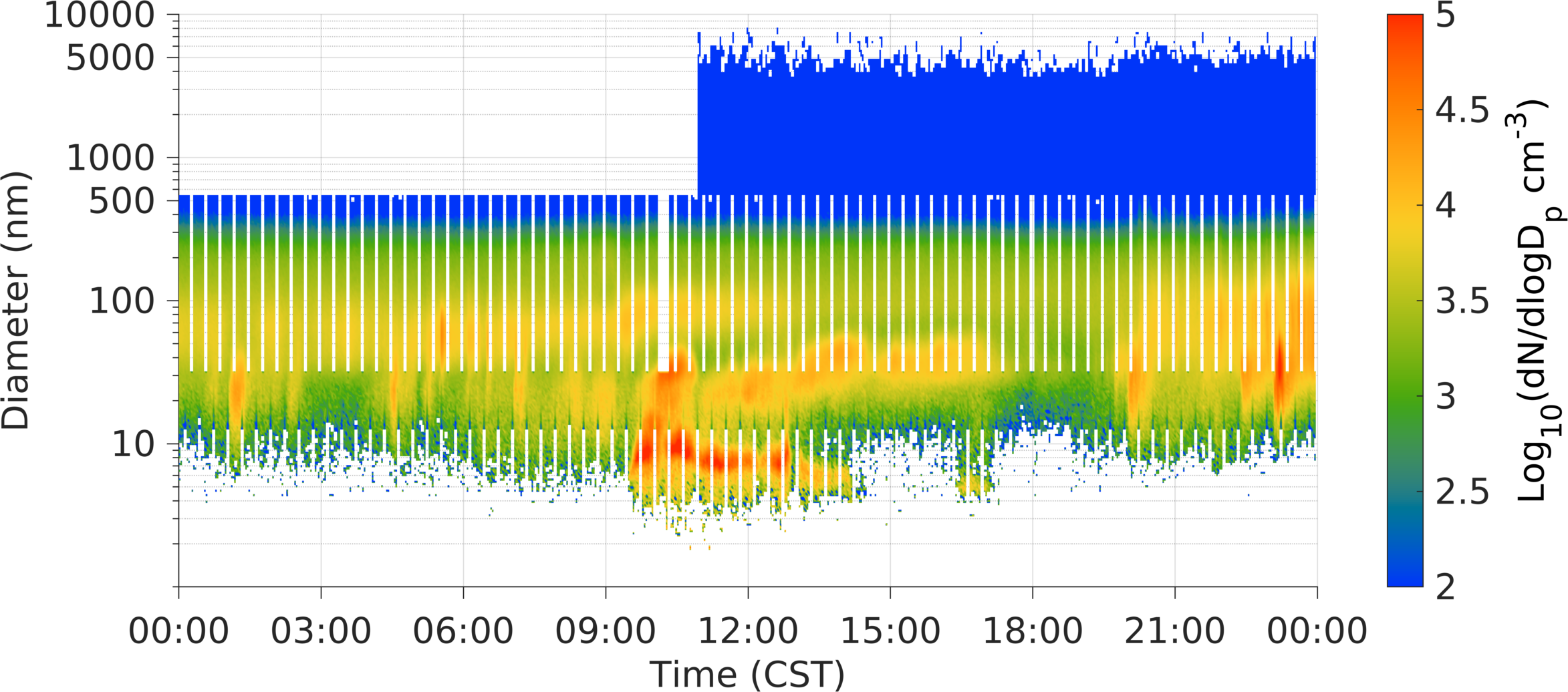
**

**S14 Fig. May 25, 2017 of 2 min particle size distribution.**

**
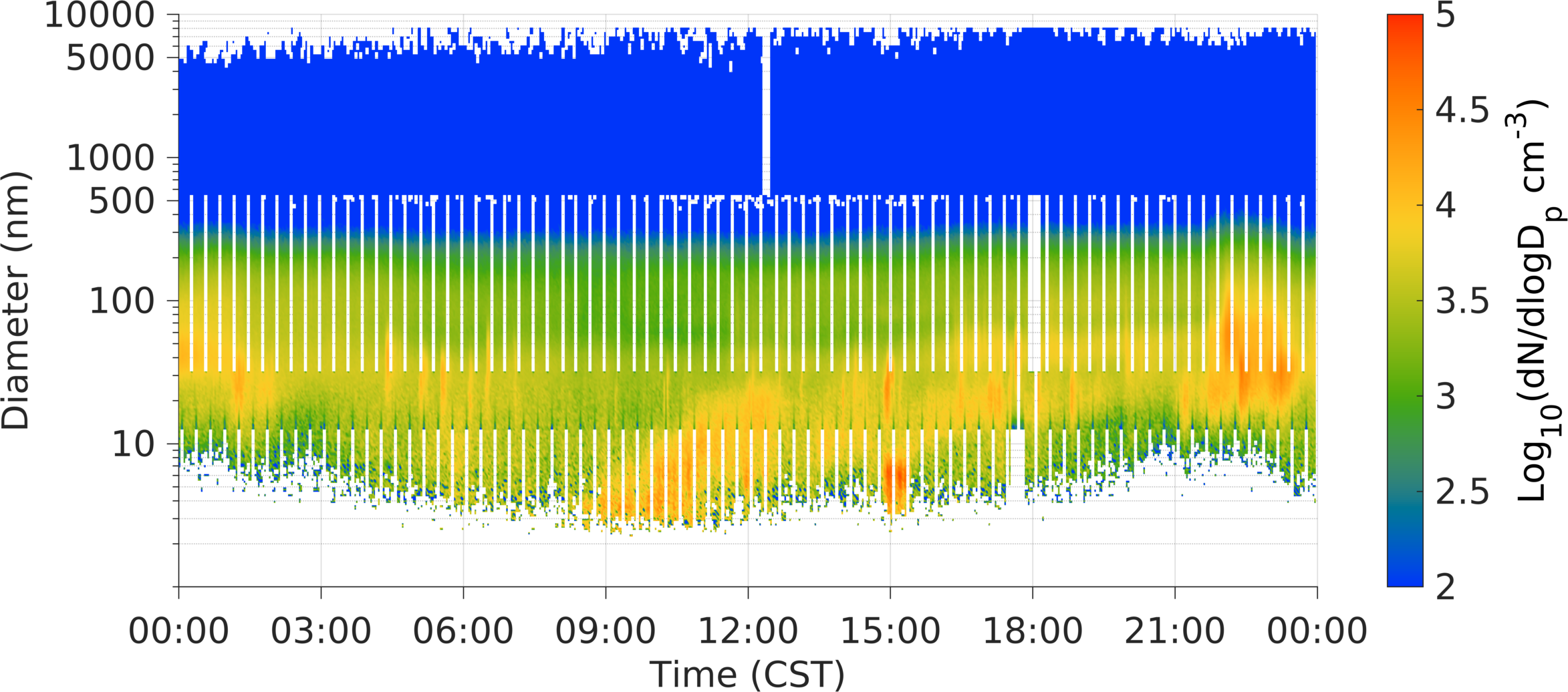
**

**S15 Fig. May 30, 2017 of 2 min particle size distribution.**

**
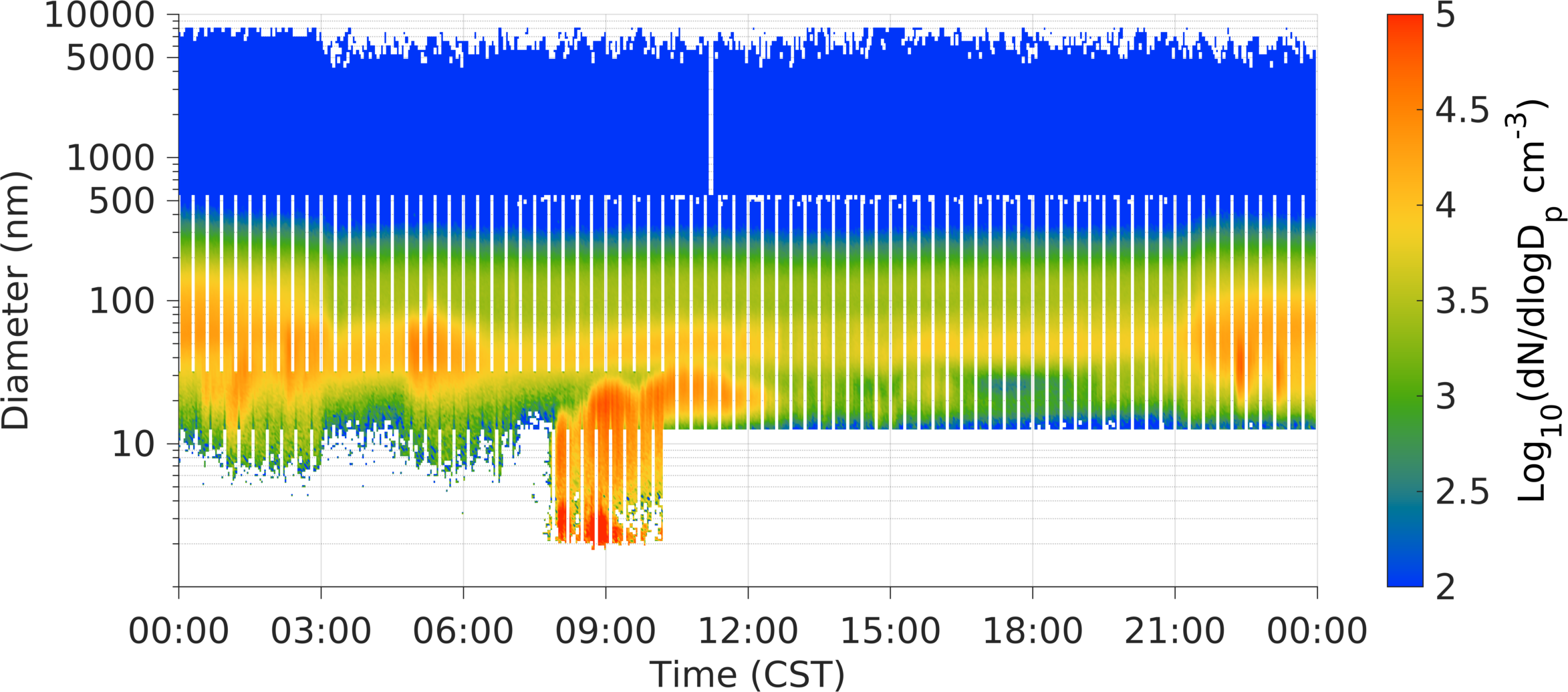
**

**S16 Fig. June 1, 2017 of 2 min particle size distribution.**

**
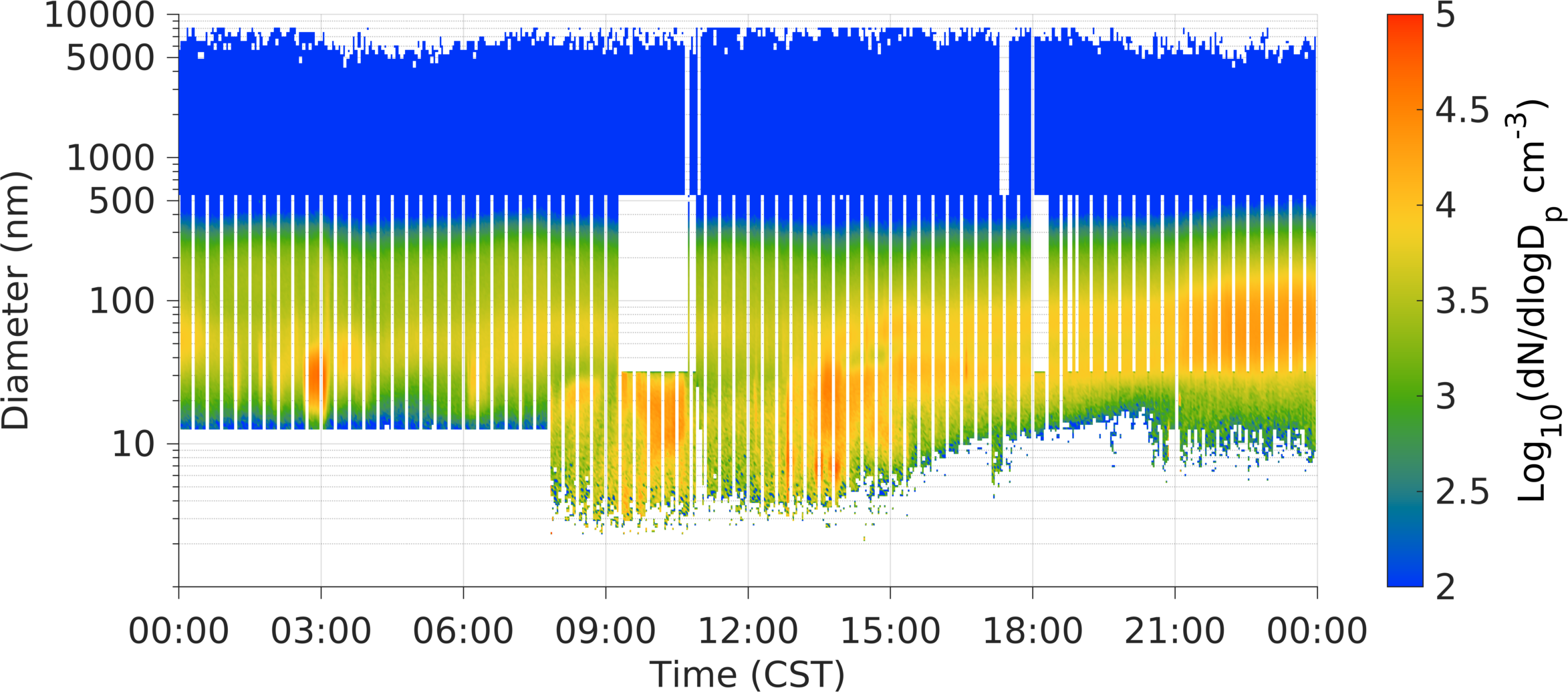
**

**S17 Fig. June 4, 2017 of 2 min particle size distribution.**

**
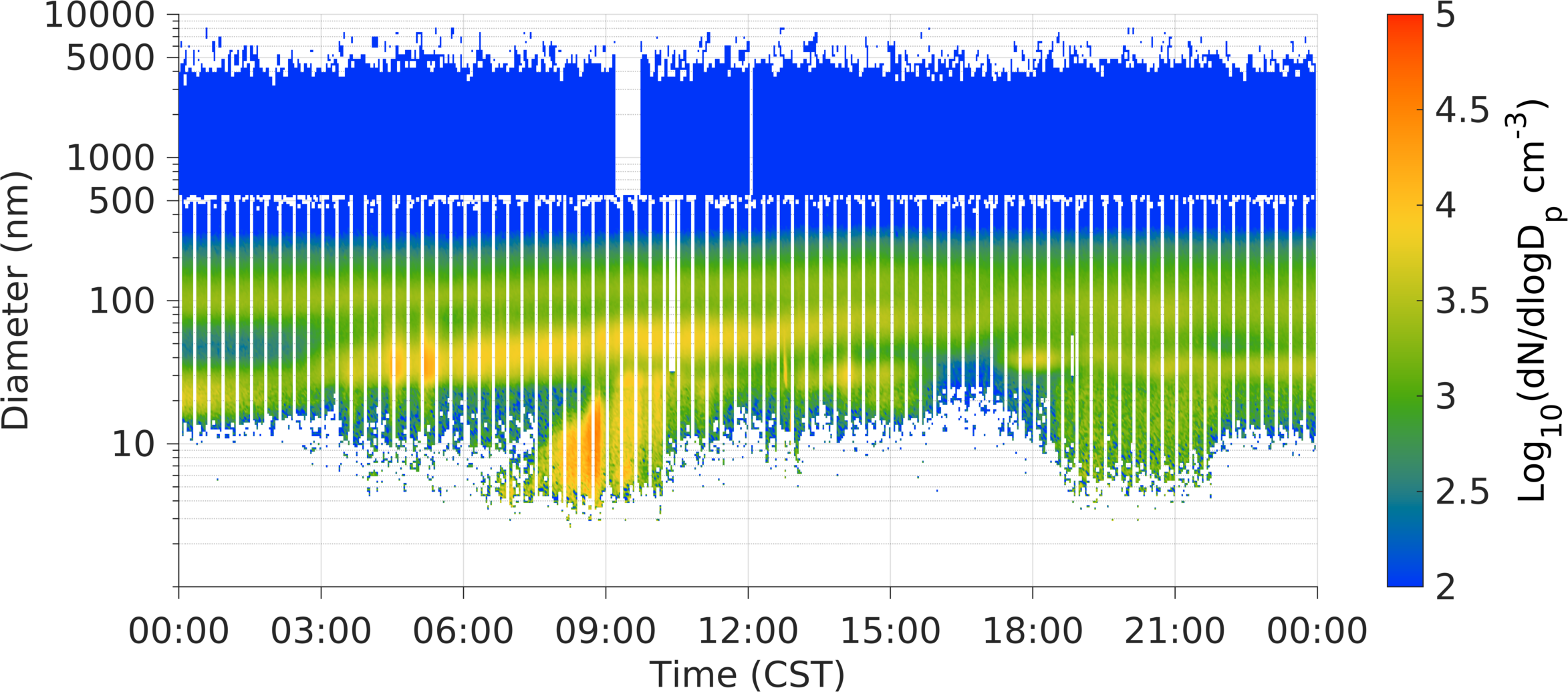
**

**S18 Fig. June 6, 2017 of 2 min particle size distribution.**

**
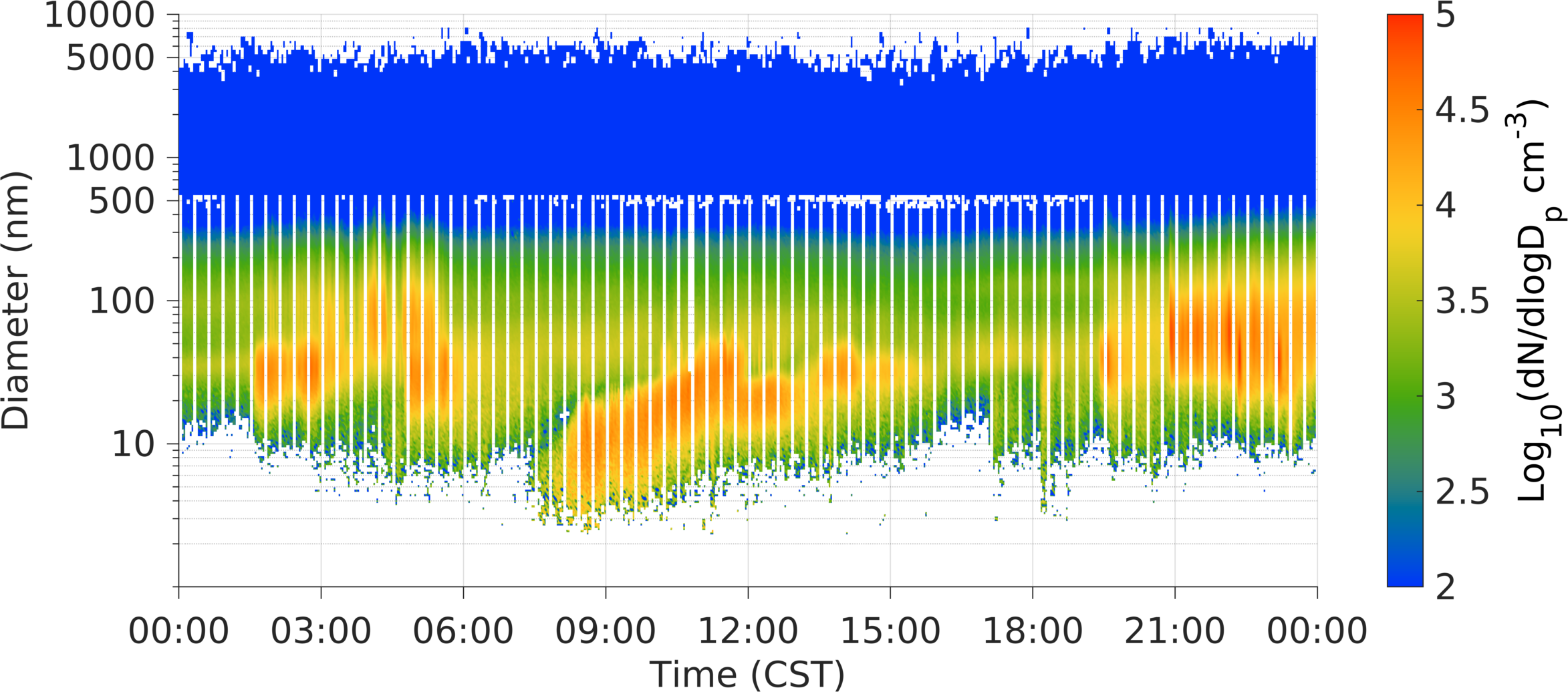
**

**S19 Fig. June 7, 2017 of 2 min particle size distribution.**

**
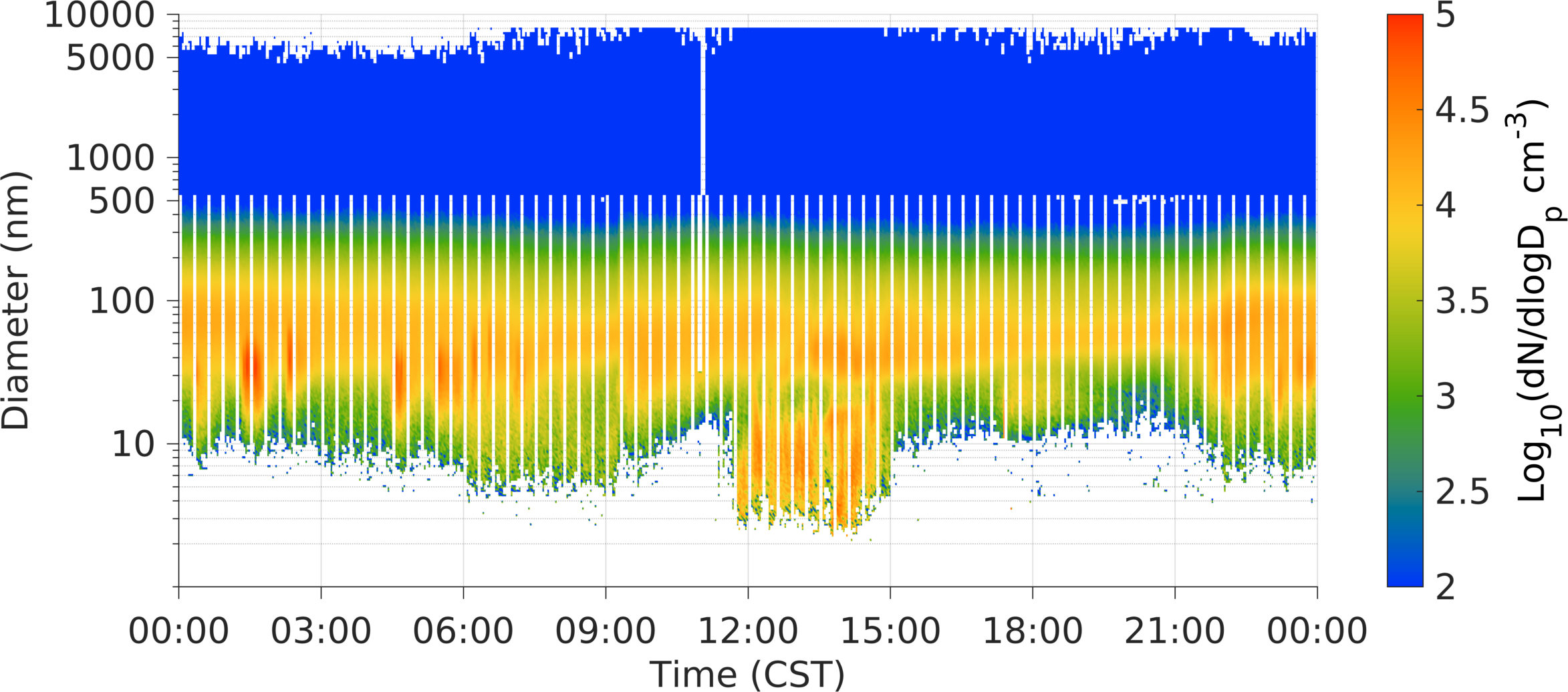
**

**S20 Fig. June 8, 2017 of 2 min particle size distribution.**


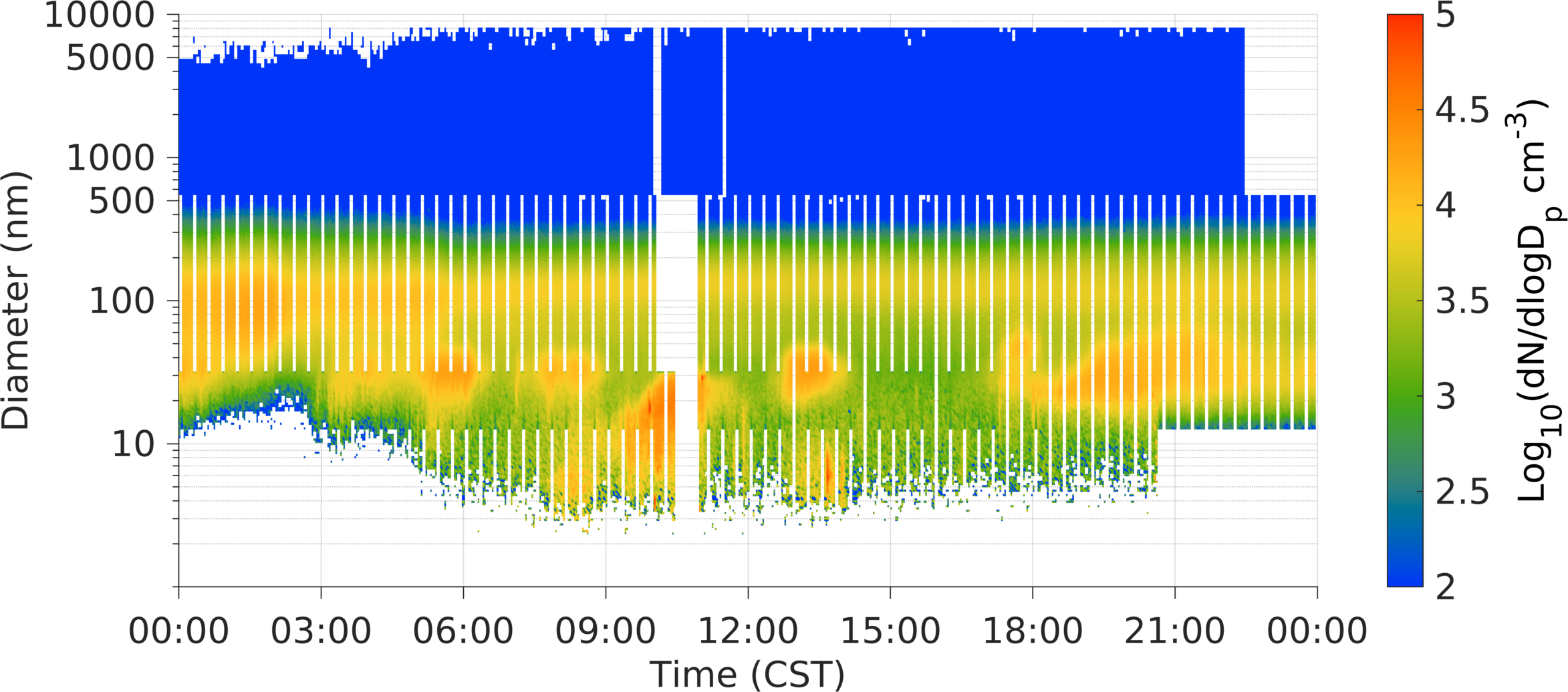


**S21 Fig. June 10, 2017 of 2 min particle size distribution.**

**
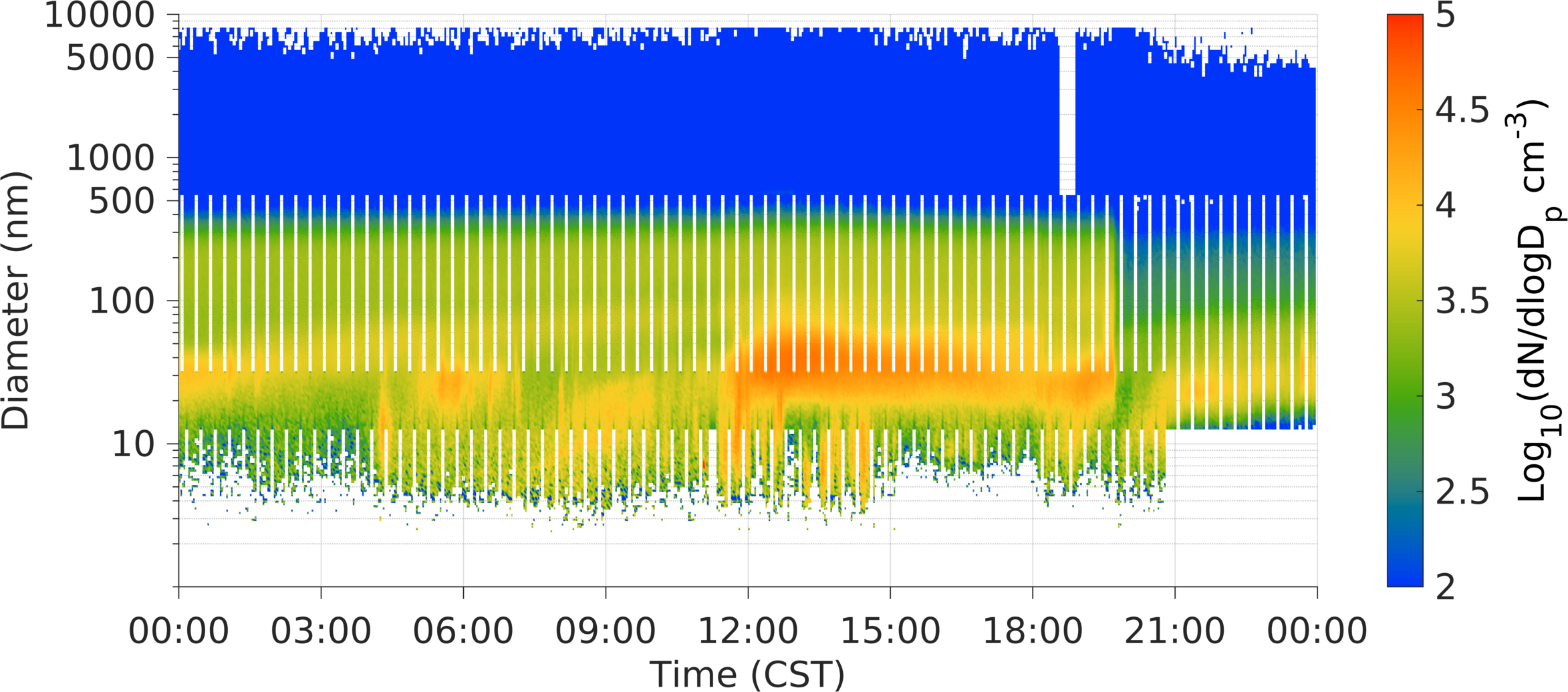
**

**S22 Fig. June 12, 2017 of 2 min particle size distribution.**

**
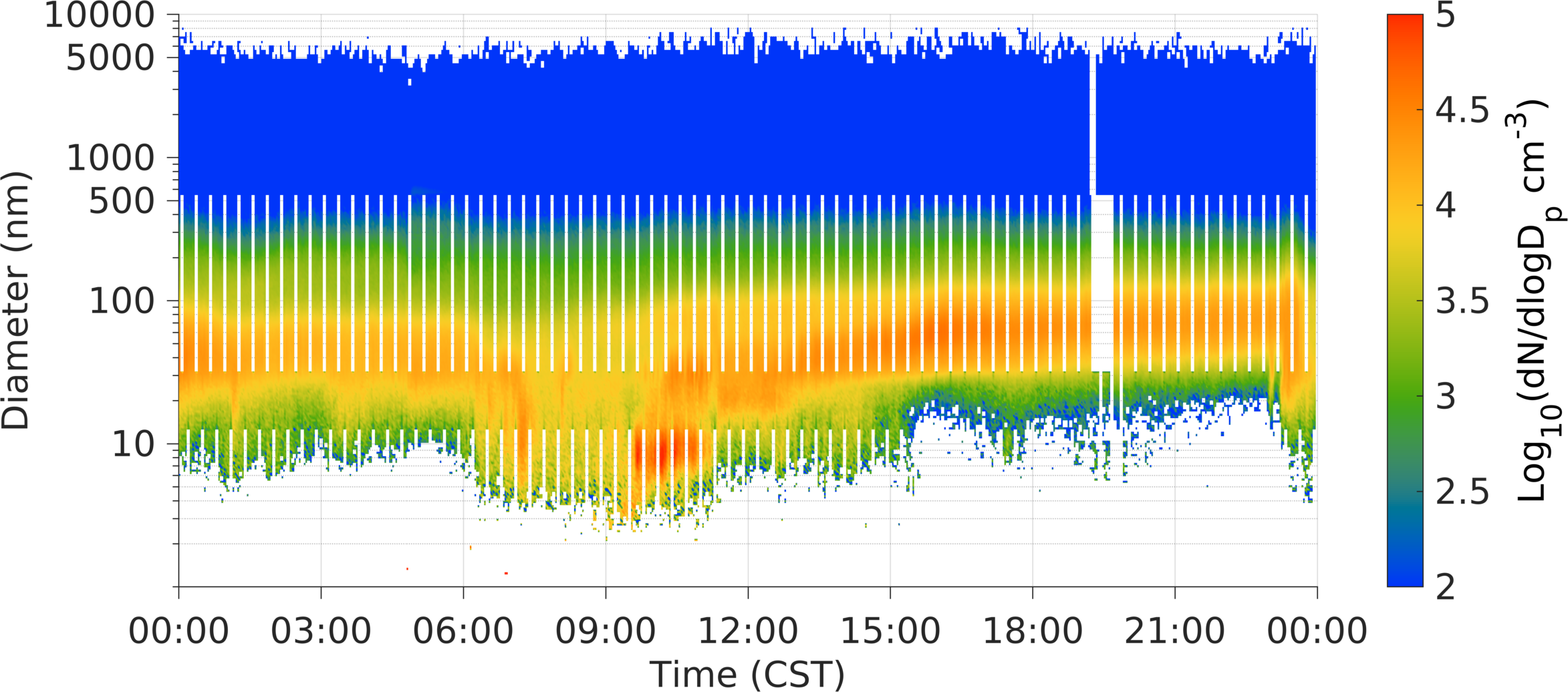
**

**S23 Fig. June 15, 2017 of 2 min particle size distribution.**

**
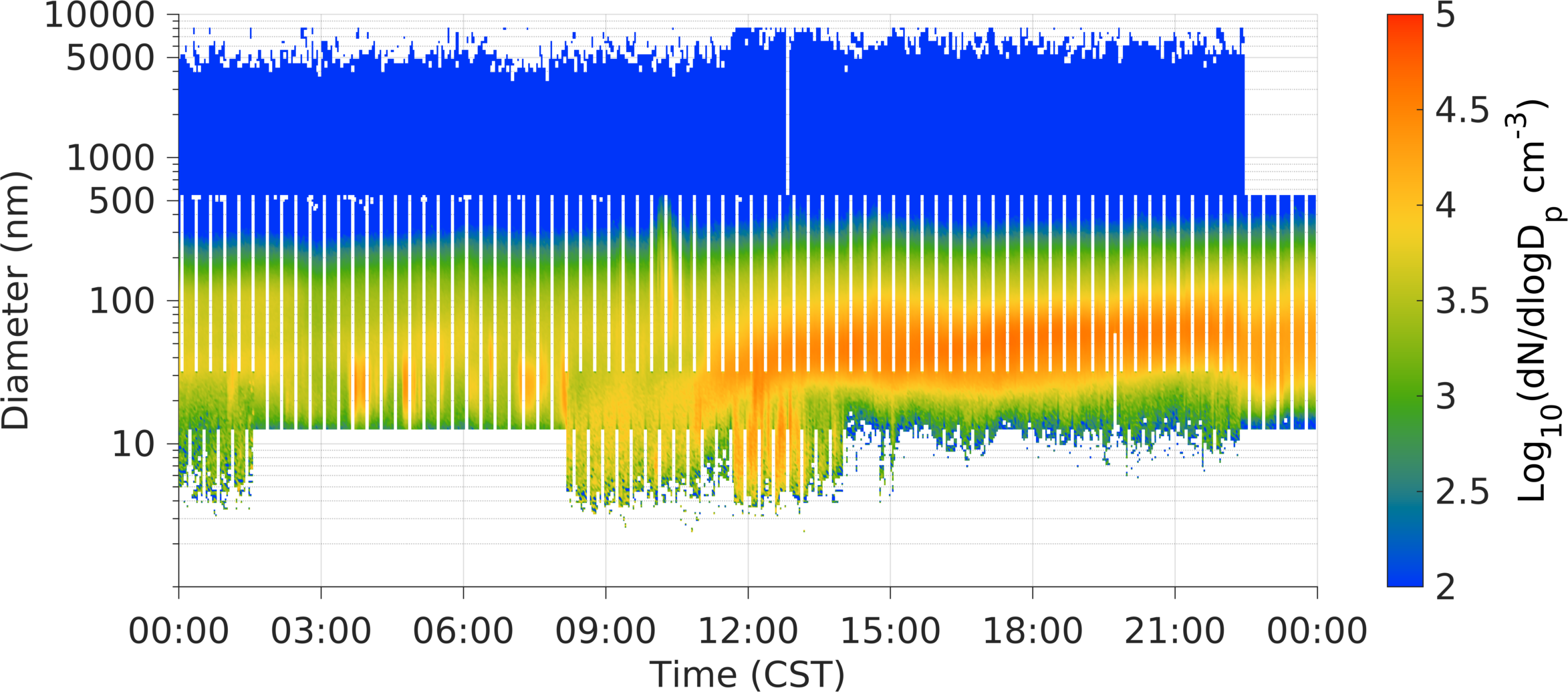
**

**S24 Fig. June 16, 2017 of 2 min particle size distribution.**

**
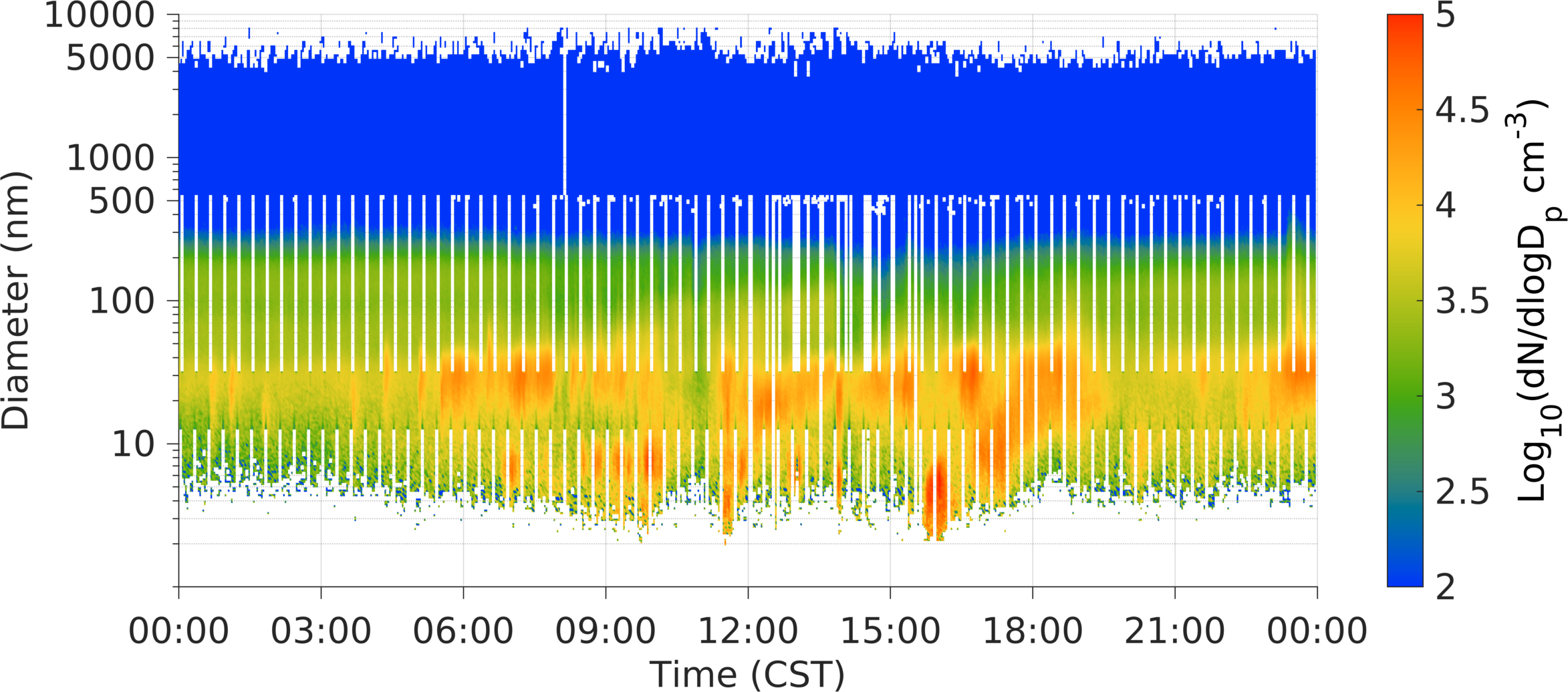
**

**S25 Fig. June 19, 2017 of 2 min particle size distribution.**
